# Delineating Calcium Signaling Machinery in Plants: Tapping the Potential through Functional Genomics

**DOI:** 10.2174/1389202922666211130143328

**Published:** 2021-12-30

**Authors:** Soma Ghosh, Malathi Bheri, Girdhar K. Pandey

**Affiliations:** 1 Department of Plant Molecular Biology, University of Delhi South Campus, New Delhi-110021, India

**Keywords:** Calmodulins (CaMs), Ca^2+^/CaM-dependent protein kinases (CCaMKs), Ca^2+^-dependent protein kinases (CDPKs), Calcineurin B-like proteins (CBLs), Glutamate-like receptors (GLRs), Cyclic nucleotide-gated channels (CNGCs), Ca^2+^-ATPases, Ca^2+^/H^+^ exchangers (CAXs)

## Abstract

Plants have developed calcium (Ca^2+^) signaling as an important mechanism of  regulation of  stress perception,  developmental cues, and  responsive gene  expression. The  post-genomic era has witnessed the successful unravelling of the functional characterization of genes and the creation of large datasets of molecular information. The major elements of Ca^2+^ signaling machinery include Ca^2+^ sensors and responders such as Calmodulins (CaMs), Calmodulin-like proteins (CMLs), Ca^2+^/CaM-dependent protein kinases (CCaMKs), Ca^2+^-dependent protein kinases (CDPKs), Calcineurin B-like proteins (CBLs) as well as transporters, such as Cyclic nucleotide-gated channels (CNGCs), Glutamate-like receptors (GLRs), Ca^2+^-ATPases, Ca^2+^/H^+^ exchangers (CAXs) and mechanosensitive channels. These elements play an important role in the regulation of physiological processes and plant responses to various stresses. Detailed genomic analysis can help us in the identification of potential molecular targets that can be exploited towards the development of stress-tolerant crops. The information sourced from model systems through omics approaches helps in the prediction and simulation of regulatory networks involved in responses to different stimuli at the molecular and cellular levels. The molecular delineation of Ca^2+^ signaling pathways could be a stepping stone for engineering climate-resilient crop plants. Here, we review the recent developments in Ca^2+^ signaling in the context of transport, responses, and adaptations significant for crop improvement through functional genomics approaches.

## 1 INTRODUCTION

### 1.1 Calcium Signaling

Calcium (Ca^2+^) is an established ubiquitous second messenger in eukaryotic systems. Plants perceive stimuli followed by an increase in intracellular Ca^2+^ levels. The cellular processes involve spatial and temporal variations in the cytoplasmic or organellar Ca^2+^ levels. The transient variations in Ca^2+^ concentrations during growth and developmental processes and responses to stress signals, are specific to a particular stimulus and are referred to as signatures. These Ca^2+^ signatures are perceived by Ca^2+^ sensors, which transduce the signal to downstream components. The Ca^2+^ signals can be decoded by factors such as cellular localization, frequency and amplitude [1-4]. Ca^2+^ flux is regulated through synergistic action involving Ca^2+^ sensors, buffers, exchangers and pumps that turn the molecular switch on-off [5]. The identification of plant gene families that encode proteins similar to Ca^2+^-permeable channels in animal systems has sparked interest in the working of Ca^2+^ channels in signaling pathways [6]. Plant Ca^2+^ sensors include calmodulins (CaMs) and CaM-like proteins (CMLs), calcineurin B-like proteins (CBLs), Ca^2+^-dependent protein kinases (CPKs/CDPKs) as well as the Ca^2+^/CaM-dependent protein kinases (CCaMKs) [7-12]. The Ca^2+^ signatures are the first layer of specificity, while the Ca^2+^ sensors and their targets make up the second layer of specificity in the signal transduction process [3, 13]. CaMs and CMLs, CBLs as well as CDPKs are the three largest families of EF-hand containing Ca^2+^ sensors. The CML and CBL families are limited only to plants, while CDPKs are also reported in protists [11].

The coding and decoding of Ca^2+^ signatures specific to the stimuli, distinct Ca^2+^ sensors, and transducers as well as plant responses, have received much attention (Table **1**). Not much is known about the functional and regulational aspects of Ca^2+^ channels and transporters along with other signaling components in plants. The recent developments in the field of genomics have shed considerable light on Ca^2+^ signaling in plants. The delineation of the entire pathway from the generation of stimulus-specific Ca^2+^ signal to the responsive gene expression is a huge challenge. Plant genomics has been used extensively for the last two decades. Extensive studies using genomic approaches have paved the way for the validation of components of Ca^2+^ signaling machinery as molecular candidates that can be utilized to overcome environmental challenges. In this review, we discuss Ca^2+^ signaling machinery and its potential for crop improvement with an emphasis on the functional genomics perspective.

## 2 CALCIUM SENSORS

### 2.1 Calmodulins, CaM-like Proteins, and Ca^2+^/CaM-Dependent Protein Kinases

CaMs are ubiquitous, eukaryotic Ca^2+^-binding proteins [7, 14]. Plant CaMs are conserved gene families with *CaMs* showing an abundant presence of introns. The largest *CaM* genes are identified in *Panicum virgatum, while* the smallest are reported in *Aquilegia coerulea*. Only 5.16% of *CaM* genes lack introns. The plant *CaM* gene family members show not more than seven introns [15]. They are acidic proteins comprising two pairs of EF-hands at both the amino (N)- and the carboxy (C)-terminals [16, 17]. They contain a conserved D-x-D motif in each of the four EF-hand domains [15]. Plant CMLs are conserved gene families, with most *CMLs* lacking introns. CML proteins are more abundant than CaMs in plants. The fourth EF-hand of CMLs contains a conserved D-x-D-x-D motif [15]. The number of *CaM* and *CMLs* family members show significant variation and do not show a correlation to the genome size [15]. Though the protein sequence of CaMs shows a high degree of conservation, gene duplication and the consequently acquired mutations result in sequence and functional divergence. Duplicated genes devoid of mutations maintain the intact protein sequence as well as function through selective pressure [18]. Ca^2+^-CaM regulated kinases (CCaMKs) are specific to plants and are involved in the regulation of several stress responses in plants [19].

Since the first cloning of CaMs in barley (*Hordeum vulgare* L.) [20], they have been identified in different plant species and implicated in various physiological functions. The CaM isoforms show variation in the targets they bind to and the subsequent regulation of the downstream components [21, 22]. CaM is encoded by seven genes, and CaM-like proteins (CMLs) are encoded by fifty genes in Arabidopsis. The numbers of EF-hands (1-6) vary in these Ca^2+^ sensors [17]. CaM isoforms vary by a few amino acid residues in plants. CaM isoforms: CaM1/4; CaM2/3/5; CaM6, and CaM7 are reported in Arabidopsis [16, 17], of which four isoforms vary by one to five amino acid residues [16]. Rice genome contains five CaM and thirty-two CML encoding genes [23]. Rice CaM isoforms, OsCaM1-1, OsCaM1-2 and OsCaM1-3, show identical sequences while OsCaM2 and OsCaM3 proteins differ by two amino acids residues [23]. Three CaM- and sixty-two CML-encoding genes have been reported in *Vitis vinifera*; VviCaMs were observed to contain four EF- hand motifs, whereas VviCMLs showed one to five EF-hand motifs. Most *VviCML* genes lack introns, while *VviCaMs* are rich in introns. These genes showed a high degree of duplication [24]. The soybean genome harbors at least two hundred and sixty-two genes that may encode proteins containing EF-hand motifs. These include six genes encoding CaMs, one hundred and forty-four genes encoding CMLs and two genes encoding CCaMKs [25]. With the utilization of genome-wide search and comprehensive studies, seventy- nine *CML* genes were identified in Chinese cabbage (*Brassica rapa* L. ssp. *Pekinensis*). These BrCMLs possessed two to four reserved EF-hand motifs. Phylogenetic studies revealed that there is a close relationship between CML homologs of Chinese cabbage and Arabidopsis [26].

Solanaceous species may have evolved a novel set of *CaM* genes in comparison with Arabidopsis and rice [27]. The genome of *Solanum pennelllii*, the stress-tolerant wild tomato, harbors six *CaM* and forty-five *CML* genes [28]. The gene families encoding CaMs in tomato, tobacco and potato genomes comprise number of highly conserved *CaM* genes. CaM protein isoforms in Arabidopsis and rice belong to a single clade while the CaMs belonging to Solanaceae form two groups, with one belonging to the clade comprising Arabidopsis and rice CaMs. This classification is based on the sequence similarity at the amino acid level and the gene structure. The genes belonging to group I comprise a single intron, whereas those belonging to group II comprise three introns, with the groups involved in distinct functions [27].

The Ca^2+^/CaM complex activates nicotinamide adenine dinucleotide kinases (NADK), which results in phosphorylation of nicotinamide adenine dinucleotide (NAD) to nicotinamide adenine dinucleotide phosphate (NADP), thus, balancing the ratio of NADP(H)/NAD(H). The derivatives of NAD^+^ and NADP^+^, cyclic adenosine 5`-diphosphate ribose (cADPR) and nicotinic acid adenine dinucleotide phosphate (NAADP), play a critical role in the regulation of Ca^2+^ homeostasis, which in turn regulate the CaM and NADK signaling hubs [29]. CaMs and CMLs do not contain a functional domain except Arabidopsis CaM7, which binds to Z-/Gbox motifs in a Ca^2+^-dependent manner as well as acts as a transcription factor in the regulation of photo-morphogenesis [30].

Intracellular Ca^2+^ as well as CaM levels regulate reactive oxygen species (ROS) homeostasis and the antioxidant machinery [31]. Plant catalases (CATs) bind CaM in a Ca^2+^-dependent manner. Unlike catalases from other organisms which do not interact with CaM, Ca^2+^/CaM stimulates the Arabidopsis CAT3 activity [32]. Ca^2+^/CaM is also activated by peroxidase from Euphorbia latex [33, 34]. CaM also regulates superoxide dismutase (SOD) in maize [35]. Exposure to CaM inhibitors exacerbates oxidative damage during heat stress in Arabidopsis seedlings [36]. The cyclase-associated actin cytoskeleton regulatory protein 1 (*CAP1*) gene encodes a CaM-regulated Ca^2+^-ATPase in maize induced specifically in the early stages of anoxia [37]. CaM may also indirectly regulate ROS machinery through the CaM-regulated gamma-aminobutyrate (GABA) synthesis as well as the GABA shunt metabolic pathway [38]. CCaMKs regulate the formation of arbuscular mycorrhiza and root nodules [19]. ZmCCaMK and OsDMI3 (OsCCaMK) from maize and rice, respectively, are involved in ABA-induced oxidative stress response [39, 40]. CaM also activates Arabidopsis mitogen-activated protein kinase phosphatase 8 (MPK8), which leads to transcriptional regulation of respiratory burst oxidase homolog D (*RbohD*) expression and suppression of wound-induced ROS generation [41]. The hydrogen peroxide (H_2_O_2_) responsive genes include those encoding CaM-binding transcription factors (CAMTAs) and co-factor (AtBTs) [12, 42, 43]. CaM and CML protein family is involved in the regulation of developmental and stress responses by transducing calcium signals into several responses at the transcriptional, post-translational, or metabolic levels. The elucidation of their interactomes holds vital cues to our understanding of stress tolerance. We have discussed their role in stress regulation in plants in the section on stress responses in biotic and abiotic stresses.

### 2.2 Ca^2+^-Dependent Protein Kinases, Calcineurin B-like Proteins, and CBL Interacting Protein Kinases

CDPKs consist of a variable domain-containing myristoylation and palmitoylation sites at the N-terminal, a Serine (Ser)/Threonine (Thr) protein kinase (PK) domain, and a CDPK activation domain (CAD). The CAD comprises a pseudo-substrate region and CaM-like domain (CLD) comprising four Ca^2+^-binding EF-hands. The pseudo-substrate region binds to the PK domain in the inactive enzyme conformation. The active enzyme conformation involves Ca^2+^- binding to the CLD and the release of the kinase domain from the bound pseudo-substrate region [44]. *CDPK* genes have evolved recently through gene duplication. The conserved D-x-D, as well as D/E-E-L motifs, have been identified in both monocot and dicot plants. The N-terminal sequence of the CDPKs shows high variability, whereas the kinase and auto-inhibitory domains are conserved [18].

CDPKs are reported in plants, including a few protozoans [45]. Genome-wide analyses for identification of CDPKs have revealed thirty-one genes in rice [46], twenty genes in wheat [47], forty genes in maize [48], thirty genes in *Brachypodium distachyon* [49], thirty-four genes in Arabidopsis [10], and forty-one genes in cotton [50]. Twenty-nine *CDPK* genes have been identified in *Solanum lycopersicum* (tomato) [51] and thirty genes in *Populus trichocarpa* (poplar) [52], while twenty-five genes have been identified in *B. napus* (canola) [53]. Soybean genome encodes fifteen genes encoding CBLs, fifty genes encoding CDPKs, thirteen genes encoding CDPK-related PKs, seventeen genes encoding Rbohs, and fifteen genes encoding unclassified proteins containing EF-hand motifs. Up to 43.1% of the EF-hand-containing genes showed differential expression under different stresses and nutritional deficiencies [25]. *In silico* analysis showed the presence of *ZmCPK* genes on all ten chromosomes in a non-random manner in *Zea mays*. Alternatively, spliced transcripts have been identified in sixteen *ZmCPKs.* The Arabidopsis and rice *CDPK* genes were also distributed on all five and twelve chromosomes, indicating that *CDPK* genes show the wide genomic distribution in plants. The maize genome has undergone duplication events with ten segmental and four tandem events. Phylogenetic analysis of *CDPK* genes in Arabidopsis, rice, and maize classifies them into four categories that may show common origin [48]. The functional diversity of CDPKs in these species might be due to variable intron frequency, thus, influencing alternative splicing as well as exon shuffling [54]. Structural analysis of *CDPK* genes in rice, maize, sorghum and Arabidopsis show that the number of introns varies from none to eighteen. *CDPK* genes contain more intron-rich regions than intron-poor regions. The expansion of multigenic *CDPK* gene family may be due to gene duplication. The genes in maize-rice showed less divergence and maximum synteny in comparison to maize-Arabidopsis as well as maize-sorghum. Minimum synteny was observed in the case of maize-Arabidopsis [55]. Higher synteny between rice and *B. distachyon* indicates high conservation between CDPK members prior to species divergence. The CDPK family in *B. distachyon* has been divided into four subgroups (I-IV) through systematic analysis. Though BdCDPKs show characteristic CDPK structure, the majority of BdCDPKs show four EF-hands, of which EF2 and EF4 exhibit high variation and divergence from those in AtCDPKs [49].

Calcineurin B-like proteins (CBL) are EF-hand-containing proteins and are encoded by 10 genes in Arabidopsis. They exhibit low sequence similarity with CaMs and may have diverged in the early stages of evolution [56, 57]. The first EF-hand of the four motifs has a Ca^2+^-binding loop comprising fourteen amino acids instead of twelve [58]. CBL interacting protein kinases (CIPKs) interact with specific CBLs. CIPKs possess a conserved structure comprising a Ser/Thr PK domain at the N-terminal, a junction domain and a regulatory domain-specific to CIPKs at the C-terminal [57]. The latter includes the autoregulatory NAF domain [Asn (N), Ala (A) and Phe (F)] that interacts with the CBLs as well as the phosphatase interaction domain (PPI) that interacts with protein phosphatases such as PP2Cs [59]. The CBL-CIPK interaction and activation involve the release of the auto-inhibitory NAF domain from the kinase domain [60], leading to cellular localization of the CBL-CIPK complexes and phosphorylation of target proteins. CBLs undergo lipid modification at their N-terminus, which determines their localization [61]. CBL-CIPK complexes are involved in the regulation of various physiological processes and stress responses [62-68].

Plant genomes contain two to fourteen *CBL* genes. The alga, *Chlamydomonas reinhardtii* and *B. rapa* contain the lowest and the highest number of *CBL* genes. Most *CBL* genes contain six to nine introns, while a few lack introns. CBL proteins contain three EF-hands and conserved motifs [69]. The *CBL* and *CIPK* gene structure are conserved among plant species [27, 69-72]. CBL and CIPK gene families may have arisen through genome duplication and diversification at the species level [72, 73]. Genome-wide studies for CIPKs led to the identification of twenty-six CIPKs in Arabidopsis [61, 74], thirty-three in rice [75], forty-three in maize [76], fifty-two in soybean [77], and twenty-seven in poplar [72]. Thirty-five *CIPK* genes were identified through a systematic genome-wide analysis of foxtail millet (*Setaria italica* (L.) Beauv) [78]. The Canola genome harbors seven *CBL* and twenty-three *CIPK* genes [73]. Cassava (*Manihot esculenta*) genome harbors eight *CBL* and twenty-six *CIPK* genes [79]. *CIPK* gene family can be differentiated based on the presence of introns. *CIPK* genes have been divided into intron-rich and intron-poor groups [74]. The expansion of CIPKs may have occurred through segmental duplication in both intron-rich as well as intron-less clades, while tandem duplication occurred in intron-less clades only [80]. Similar observations have been reported in *CIPK* genes identified in both monocots as well as dicots, like maize, rice and poplar [72, 74, 77, 80]. The seventy-eight and eighty genes in *Gossypium barbadense* and *G. hirsutum* are characterized by the presence of exons based on phylogenetic analysis as well as gene structure. The expansion of the *Gossypium CIPK* gene family may be due to segmental duplication as well. Their expression patterns indicate spatial as well as temporal specificity. Seventeen non-synonymous single nucleotide polymorphisms (SNPs) have been observed in fourteen *GhCIPK* genes that co-localize with quantitative trait loci (QTLs) pertaining to oil/protein content [81]. CIPK encoding genes in cotton are closely related to those of Arabidopsis and poplar [82]. The grapevine genome harbors eight CBL and twenty CIPK encoding genes. The synonymous and non-synonymous substitution rates of CBL and CIPK gene families indicate that VvCBL encoding genes may be more conserved in comparison to VvCIPK encoding genes [72]. CBL-CIPK interactions constitute a major part of the signaling modules involved in the regulation of stress responses which are counteracted by the action of protein phosphatases (PP). The role of genes encoding CDPKs, CBLs and CIPKs as critical components in stress regulation is highly valued and has been studied in different crops.

## 3 CALCIUM FLUX: REGULATION THROUGH CHANNELS AND TRANSPORTERS

The [Ca^2+^]_cyt_ maintenance is regulated by (i) Ca^2+^-permeable cation channels that facilitate a passive influx of Ca^2+^ in sync with the electrochemical gradient; (ii) Ca^2+^-ATPases and Ca^2+^/H^+^ exchangers that transport Ca^2+^ against the electrochemical gradient; and (iii) intracellular compounds that buffer [Ca^2+^]_cyt_. Plant Ca^2+^ transporters are categorized as hyperpolarization-activated Ca^2+^ channels (HACC); depolarization-activated Ca^2+^ channels (DACC) and voltage-independent Ca^2+^ channels (VICC) [83]. Ca^2+^ channels could be rapidly activating (with complete activation time in milliseconds), slowly activating (with complete activation time in seconds), and ‘spiky’ burst-like channels or single-channel events in the whole-cell mode [83, 84]. Some Ca^2+^ channels are dependent on voltage alone for activation and are termed ‘constitutive’ as opposed to ligand-gated Ca^2+^ channels that are dependent on particular ligands for activation. The extracellular ligands include amino acids, ROS and purines, while the intracellular ligands include phosphorylated sugars, H_2_O_2_, lipid derivatives, 3’,5’-cyclic adenosine monophosphate (cAMP), 3’,5’-cyclic guanosine monophosphate (cGMP), cADPR and phosphate. The channel activities are regulated through modulators such as cytosolic Ca^2+^, H^+^, ROS and polyamines [83]. Most plant Ca^2+^ channels are non-selective cation channels (NSCCs). They are significantly permeable to mono- as well as divalent cations (K^+^, Na^+^, NH_4_^+^, Cs^+^, Rb^+^, Ba^2+^, Sr^2+^) and are weakly permeable to some ions such as Zn^2+^, Mn^2+^ and Mg^2+^ ions [85].

The main Ca^2+^ reserves are the vacuoles, endoplasmic reticulum (ER), mitochondria, plastids, and apoplast. Membrane channels regulate Ca^2+^ influx from the storage compartments towards cytosol [1]. Channels allow passive movement of Ca^2+^ in the cytosol according to their electrochemical potential gradient [86]. Five families of Ca^2+^ channels that regulate the influx have been reported in Arabidopsis: cyclic nucleotide-gated channels (CNGC), glutamate-like receptors (GLRs), two pore channels (TPCs), mechanosensitive channels and reduced hyperosmolality-induced [Ca^2+^]_cyt_ increase channels (OSCAs) [87-92]. The transporters localized mostly in the tonoplast and plasmalemma allow accumulation of Ca^2+^ in vacuoles and apoplast, respectively, against the electrochemical potential gradient. The channel and transporter activities require stringent regulation to maintain the [Ca^2+^]_cyt_ levels. Ca^2+^ transport against its electrochemical potential gradient from the cytosol to organellar storage compartments or apoplast, is regulated by Ca^2+^ ATPases and H^+^/Ca^2+^ antiporters/exchangers [86]. The P-type Ca^2+^-ATPases hydrolyze ATP as an energy source and the Ca^2+^/H^+^ exchangers of the CAX family (Ca^2+^/cation exchangers) utilize the proton gradient across the membranes towards the cytosol [86, 93, 94]. Ca^2+^ transporter families that regulate efflux in Arabidopsis are: autoinhibited Ca^2+^-ATPases (ACAs), ER-type Ca^2+^-ATPases (ECAs), P1-ATPases, Ca^2+^ exchangers (CAX), CCX (cation/Ca^2+^ exchangers), Na^+^/Ca^2+^ Exchanger (NCX) protein and the mitochondrial calcium uniporter complex (MCUC) [95-99]. Phylogenetic analysis has shown the conserved evolution of genes encoding Ca^2+^ transporters in monocots as well as eudicots except for group IV GLRs in rice [100]. The subcellular localization of Ca^2+^ channels and transporters are represented in Fig. (**1**).

### 3.1 Glutamate-Like Receptors

Glutamate-like receptor (GLR) functions as an integral membrane protein that exhibits a ligand-gated ion channel activity [101]. GLR encoding genes exhibit major divergence in both plants and animals. Up to 20 such genes have been identified in Arabidopsis, while fifteen genes have been identified in the tomato genome [102-104]. Rice genome encodes twenty-four GLRs [100]. The soybean genome contains thirty-five putative GLR genes and may contain three to eight transmembranous domains. In addition, GLR proteins contain a ligand-gated ion channel and most of these possess a receptor family ligand-binding domain. Phylogenetic analysis of GLRs in Arabidopsis, rice and soybean indicate a divergence during the evolution of GLR family across different species [105]. Angiosperm genomes harbor a number of *GLR* genes, this has been a limitation in the identification of prominent phenotypes [106]. GLRs show ubiquitous expression in plant tissues [107].

Plant GLRs are involved in physiological functions like stomatal opening, carbon/nitrogen balance, lateral root formation, pollen tube growth, and defense responses [108]. GLRs regulate Ca^2+^ influx across the plasma membrane and apical [Ca^2+^]_cyt_ gradient, thus, influencing the growth and morphogenesis of pollen tube in Arabidopsis and tobacco. The *Atglr1.2* and *Atglr3.7* knock-out mutant plants exhibited male reproductive phenotypes with a reduced number of seeds per silique. The *Atglr3.7-1* pollen tubes showed slower growth while the *Atglr1.2-1* pollen tubes have deformed tips and tubes [109]. GLR1 and GLR2 are involved in sperm chemotaxis and transcriptional regulation in *Physcomitrella patens.* They are required in the induction of the expression of a sporophyte-associated BELL1 transcription factor homolog necessary for zygotic development [106].

The *GLR3.3* and *GLR3.6* knockout mutants do not exhibit an increase in the glutamate-induced [Ca^2+^]_cyt_ levels [110, 111]. AtGLR3.6 is involved in primary and lateral root development in Arabidopsis. The loss-of-*AtGLR3.6* function resulted in a decrease in primary root growth, number of lateral roots and root meristem size. They also showed upregulation of Kip-related protein 4 (KRP4) encoding gene, a cell- cycle related gene that controls plant`s growth in response to exogenous hormones [112] and a decrease in [Ca^2+^]_cyt_ levels. AtGLR3.6 overexpression showed a downregulation of *KRP4* gene and induction of primary as well as lateral root growth, thus, implicating *AtGLR3.6* in the development of root system through manipulation of *KRP4* expression [111]. The *glr3.7-2* mutant seeds showed lower elevations in [Ca^2+^]_cyt_ levels and enhanced sensitivity to salt stress during germination. The *GLR3.7-S860A*-OE lines also exhibited lower sensitivity to salt stress during primary root growth, thus, implicating *GLR3.7* under salinity stress in Arabidopsis [113]. The variation in GSH (reduced glutathione)-induced [Ca^2+^]_cyt_ levels exhibited sensitivity to GLR antagonists and diminished in *atglr3.3* mutant plants. They also exhibited an increase in susceptibility to *Pseudomonas syringae* pv. tomato (*Pst*) DC3000 [114].

### 3.2 Cyclic Nucleotide-Gated Channels

Cyclic nucleotides bind the GAF (cyclic GMP, adenylyl cyclase, FhlA) and the CNB (cyclic nucleotide binding) domains in proteins [115]. The CNB domain is reported in CNGC and the Shaker-type potassium channels [115, 116]. CNGCs are tetrameric ion channels that are regulated by binding cyclic nucleotides. They are structurally similar to the ‘Shaker-like’ pore-loop ion channels in plants [117, 118]. The CNGCs exhibit lower ion selectivity in comparison to the Shaker-type K^+^ channels and allow monovalent cations like K^+^ and Na^+^ and divalent cations like Ca^2+^ [119]. The channel complex is tetrameric, with each subunit comprising six transmembrane α-helices, S1-S6, with both N- and C-terminals oriented towards the cytosol. The pore loop region (P-loop) occurs between the S5 and S6 domains. It includes the pore helix as well as the selectivity filter. The CNB domain overlaps with a calmodulin-binding site (CaMBS) and is located at the C-terminal [120]. The CaMBS includes Isoleucine-glutamine (IQ) domains, which are present in all CNGCs [118, 121]. The unique selectivity filters comprising of the triplet of amino acids located in the P-loop have been identified in plant CNGCs using structural modeling. The GQN, GQG, AGN, GQS, AND, and GN-L triplets are plant CNGC selective filters [119, 122]. The CNGCs that possess the AND, GQN, GQG and GQS filters facilitate Ca^2+^ uptake [83].

Genome-wide identification of CNGC gene family members has been pursued in different plant species. Twenty CNGCs have been reported in Arabidopsis [ 123 ], seventeen and twenty-one genes have been reported in rice and pear, respectively [ 100, 124] and eighteen genes have been identified in tomato [125]. *P. trichocarpa* harbors twelve [116] and moss has eight CNGC encoding genes [126]. The soybean genome contains thirty-nine *CNGC* genes including fifteen segmentally as well as five tandem duplicated genes [105].

CNGCs are involved in the growth of pollen tubes, nutrition, Ca^2+^ homeostasis, transport of divalent heavy metal ions, plant-pathogen interactions, cell death as well as tolerance to salinity, cold and drought stresses [117, 119]. The AtCNGC5 and AtCNGC6 are cGMP-dependent Ca^2+^-channels in the guard cell plasma membrane [127], while CNGCs 7 and 8 are prominent in the plasma membrane at the growing pollen tube flanks [128]. AtCNGC10 localizes at the plasma membrane of mesophyll as well as parenchyma cell [129]. CNGC19 and CNGC20 localize in tonoplast [130].

Antisense *AtCNGC10* lines showed early flowering, high starch levels in chloroplasts, slow response to gravitropism, decrease in palisade cell length, thickness and surface area of leaves [129]. CNGC18 is prominent in the pollen tube tips [131]. The *cngc18* mutants exhibited defective pollen tubes rendering them short, kinky, mostly thin, and prone to bursts after brief growth [131]. AtCNGC18 is involved in the regulation of Ca^2+^ influx towards the establishment and maintenance of the Ca^2+^ gradient. This facilitates the formation and growth of the tips of pollen tubes. In addition, it is involved in directing the pollen tube to ovules through its transmembrane domains [132]. CPK32 was identified through a genome-wide survey for the CPKs involved in pollen tube growth. *CPK32* overexpression resulted in extreme depolarization in pollen tube growth and the consequent bulging at the tip [133]. The polar growth of the short, bulbous pollen tubes of the *CPK32*-OE lines accumulated high Ca^2+^ levels in the tip and showed growth arrest. The co- expression of *CNGC18* with *CPK32* resulted in a synergistic effect on major depolarization of pollen tube growth. CNGCs activation on co-expression with a CPK32 indicates phosphorylation-regulated mechanism. Ca^2+^-activated CPK32 interacts with and activates CNGC18, thereby elevating Ca^2+^ levels in the polarized pollen tube growth [134].

CNGCs are also involved in the symbiotic responses in *Medicago truncatula*. The Ca^2+^-permeable CNGCs, localized in the nuclear envelope, are complex with the K^+^-permeable channel, thus, regulating the nuclear Ca^2+^ release. CNGCs 15a, b, and c are involved in the formative stages of rhizobial as well as mycorrhizal symbiotic associations of *M. truncatula*. The *cngc15a*, *cngc15b* and *cngc15c* mutants exhibited a decrease in nodulation, colonization by *Rhizophagus irregularis*, impaired rhizobial growth as well as activation of early gene expression in response to *Sinorhizobium meliloti* or *R. irregularis* through lipochitooligosaccharide (LCO) signaling [135].

### 3.3 Two-Pore Cation Channel

Two-pore channels (TPCs) are members of the voltage- gated ion channel superfamily and show ubiquitous expression in plants and animals [136, 137]. TPCs are localized to the vacuoles in plants [138] while they localize to the vesicles of the endosomal and lysosomal system in humans [139]. Depolarization-activated slow vacuolar (SV) channels comprise the dominant conductance at micromolar [Ca^2+^]_cyt_ levels. The *TPC1* gene of Arabidopsis encodes an SV channel [138]. Rice genome also harbors a single *OsTPC1* gene [100]. TPC orthologues, *GmTPC1* and *GmTPC2* genes containing twenty-three introns, have been identified in the soybean genome. GmTPC1 and GmTPC2 proteins contain 738-739 amino acid residues and contain twelve to thirteen trans-membrane domains. Both proteins are closely related to the AtTPC1 and OsTPC1 proteins and contain ion transport protein domain as well as EF-hand pair [105].

TPC comprises two homologous domains, each made of six transmembrane helices and one pore [140]. Plant TPCs have two cytosolic EF-hand domains, which possess Ca^2+^-receptor characteristics and act as structural sites that facilitate channel responses during physiological changes in Ca^2+^ levels [141]. The activation of AtTPC1 is dependent on voltage as well as cytosolic Ca^2+^ [142]. The AtTPC1 contains voltage-sensing domains, VSD1 and VSD2 [142]. VSD2 primarily mediates voltage sensing [143]. AtTPC1 is selective for Ca^2+^ than Na^+^, although it is non-selective for monovalent cations such as K^+^, Li^+^ and Na^+^ ions [144]. TPC activation occurs due to a low transmembrane potential [142, 145] and a rise in [Ca^2+^]_cyt_ levels [142]. TPC inhibition occurs due to low luminal pH and Ca^2+^ levels [143]. Phosphorylation sites in the C- and N-terminal domains allosterically modulate the activation of [Ca^2+^]_cyt_ levels [143]. Plant as well as human TPCs are phosphoregulated [145, 146].

The *Attpc1* knockout mutant plants show an absence of functional SV channel activity, with defective ABA-induced suppression of germination as well as stomatal responses to extracellular Ca^2+^ [138]. TPC1 is involved in the stomatal closure, maintenance of differential K^+^ and Ca^2+^ storage in the epidermal and mesophyll tissue, extracellular Ca^2+^ perception, wounding as well as JA signaling [138, 140, 145, 147]. TPC1 channels are involved in systemic Ca^2+^ response to insect herbivory in leaves [148]. They are also involved along with GLR3.3, GLR3.6, and AtRBOHD-generated ROS-assisted Ca^2+^-induced Ca^2+^ release in the roots in response to salinity stress [149-151]. TPC1 channels may function as oxygen sensors in plants under water logging conditions [152].

### 3.4 Annexins

Annexins are a multigenic, conserved superfamily of cytoplasmic proteins that associate with phospholipids in the membrane in a Ca^2+^-dependent or -independent manner. They are expressed in protists, plants and animals. Plant annexins comprise repetitive annexin domains with Ca^2+^-binding conserved endonexin fold [153]. Their protein structure contains four repeats (I-IV) of 70 amino acids approximately and each of these may contain a type II Ca^2+^-binding bipartite motif. This motif is highly conserved in repeat I, with moderate conservation in repeat IV in plant annexins [154]. Arabidopsis genome harbors eight genes that encode annexins; wheat and barley genomes harbor 25 and 11 genes, respectively [155]. The soybean genome contains twenty-six *ANN* genes, *GmANN1*-*GmANN26*, with the GmANN proteins containing 141 to 362 amino acid residues [105]. Rice genome harbors ten *ANN* genes [100]. The maize genome contains not more than five *ANN* genes, whereas Brachypodium contains one *ANN* gene [156]. *ANN* genes are highly conserved in Brassicaceae species. *B. rapa*, *B. oleracea*, and *B. napus* harbor thirteen, twelve and twenty six *ANN* genes, respectively [157].

Annexin encoding genes show conserved exon-intron positions with variability in intron number among the different organisms. Gene duplication may have caused the divergence as well as expansion of annexin gene families [156]. The nucleotide diversity of *ANN1-12* genes among *Triticum aestivum*, *Triticum urartu, Aegilops tauschii*, and *Hordeum vulgare* was observed to be very low. *ANN2* gene has been observed to be under positive selection, whereas *ANN6* as well as *ANN7* have been observed to be under purifying selection in the four species [155]. The number of introns in each *ANN* gene range from 0 to 8 in Viridiplantae. Annexins from soybean and grape vine showed not more than eight introns [156]. The loss of introns may be predominantly involved in the evolution of *ANN* genes in land plants. The loss of introns in Arabidopsis, as well as rice, is 12.6 and 9.8 times, respectively common as compared to the gain of introns [158]. The first exon of phase 1 is flanked by an intron, whereas the remaining exons occur in phase 0 at the exon-intron junctions in annexin genes in land plants. The positions of introns in phylogenetic groups, as well as their conserved phases with symmetric exons, suggest a common ancestry of annexin encoding genes in the land plants. The conservative intron phases may have imparted stability in the evolutionary process as in the case of vertebrate annexins [156, 159]. Alternative splicing may be predominant in case of annexins in monocots except for sorghum. *In silico* analysis suggest that the *Os09g23160* and *Os02g51750* primary transcripts of rice annexins may generate additional transcripts [9]. The Arabidopsis annexin *ANNAt2* may generate transcripts as well [156].

Annexins are involved in the regulation of abiotic and biotic stress responses in plants [153]. Native maize annexins may act as multifunctional proteins with peroxidase activity that lead to elevation of [Ca^2+^]_cyt_ levels as well as passive Ca^2+^- and K^+^-permeable conductance directly [160]. Arabidopsis annexins harbor phosphosites and associate with other proteins such as kinases [161]. Arabidopsis annexin1 (AtANN1) mediates the OH^•^-activated Ca^2+^- and K^+^-permeable conductance in the root epidermal plasma membrane. AtANN1 also responds to high NaCl levels by regulating ROS-activated Ca^2+^ influx. The loss-of-function *Atann1* mutants do not show NaCl-activated Ca^2+^ influx, adaptive signaling as well as the salt-responsive formation of secondary roots, implicating AtANN1 in the cellular adaptation to salinity stress in roots [162]. ANN1 and ANN2 are involved in post-phloem sugar movement to the root tip thereby affecting the rate of photosynthesis in cotyledons. The *ann* mutant seedlings exhibited a reduced growth of primary root and alterations in columellar cells in root caps in the absence of sugar. The primary roots showed high sugar levels, with the root tips exhibiting impaired post-phloem sugar movement and the cotyledons exhibiting an upregulation of photosynthetic gene expression and chlorophyll levels [163].

ANN5 plays an important role in pollen development, germination as well as the growth of pollen tubes through Ca^2+^-mediated endomembrane trafficking [164]. The *ANN5*-RNAi (RNA interference) lines exhibited abortive pollen phenotype characterized by small, mis-shaped and collapsed pale-green pollen, correlating with the down-regulation of Ann5 function [165]. The Arabidopsis *ANN5*-RNAi lines exhibited sterile pollen phenotype while the pollen germination, growth of pollen tubes, and cytoplasmic streaming showed more resistance to Brefeldin A (BFA) in *ANN5*-OE lines [164]. In addition to the defects in pollen development, ANN5 plays an important role during the transitory phase between the vegetative and generative stages as well as in embryonic development and embryonic growth. *ANN5-*RNAi lines exhibited aberrations during these phases that resulted in delayed bolting time and smaller embryo size [166].

### 3.5 Mechanosensitive Channels

Mechanosensitive channels regulate Ca^2+^ signals in response to membrane tension and mechanical osmotic stress in plants. These include mechanosensitive-like channels (MSLs), Mid1-complementing activity channels (MCAs), and two-pore potassium (TPK) families [167]. Arabidopsis genome harbors 10 genes encoding MSLs, two genes encoding MCAs, and one gene encoding mechanosensitive piezo channels [167-169]. Sixteen genes have been identified in the soybean genome that may encode putative MSL proteins. These proteins contain 387-947 amino acid residues and may contain three to seven trans-membrane domains as well as a mechanosensitive ion channel domain [105]. *Arabidopsis* MCA1 and MCA2 form homotetrameric complexes. Both these channels are single-pass type I integral membrane proteins that have extracellular N termini and intracellular C termini. These channels have an EF hand-like motif, coiled-coil motif and plac8 motif. MCA1 and MCA2 comprise 421 and 416 amino acid residues, respectively, with the two sharing 73% identity. Both are involved in the regulation of Ca^2+^ influx [170, 171]. Five potential *MCA* gene loci, *GmMCA1* to *GmMCA5,* containing five to eight introns, have been identified in the soybean genome. The encoded proteins contain 403-418 amino acid residues. The highly conserved MCA protein sequences contain a putative trans-membrane domain as well as a Cys-rich domain known as the PLAC8 motif. The GmMCA proteins are closely related to the AtMCA1, AtMCA2, and OsMCA1 proteins [105].

MSL proteins localize at plastid, plasma membrane and ER except for MSL1, which localizes at mitochondria [167]. MSLs in mitochondria and chloroplast comprise five transmembrane domains, with the fifth domain acting as a pore region, whereas the voltage sensor encoding domain is absent in MSLs. MSLs located in the PM (MSLs 8, 9 and 10) are tetramers that comprise six transmembrane domains. The sixth transmembrane domain acts as the pore region [83]. MSL1, an integral mitochondrial membrane protein, is involved in ion transport resulting in loss of potential of the mitochondrial membrane at high levels as well as maintenance of redox homeostasis during abiotic stress. The loss-of-function of *MSL1* resulted in a raised oxidation state of the glutathione pool in mitochondria under moderate heat as well as heavy metal stress conditions [172]. Arabidopsis MSL10 expressed in *Xenopus laevis* exhibited a preference for anions [173].

MSL2 and MSL3 are required to overcome the hypo-osmotic stress on chloroplasts during normal growth conditions. The plastids in the leaf epidermis of *msl2-1msl3-1* mutant plants exhibited rapid and reversible alterations in volume as well as shape under extracellular osmotic stress. The leaf epidermal plastid phenotype of *msl2-1msl3-1* mutant plants was restored by enhancing cytoplasmic osmolarity, supply of growth media with osmolytes, or withholding water resulting in plastids similar to those in wild type [174]. The *msl2* phenotype includes not only defects in chloroplast morphology as well as function but also in leaf morphology, starch accumulation and seedling viability [175]. MSL2 and MSL3 are involved in the chloroplast division machinery. Along with the components of the Minicell C (MinC) system, they are involved in the regulation of size of chloroplast and the formation of Filamentous temperature sensitive Z (FtsZ) ring. The *msl2msl3* double mutant plants exhibited enlarged chloroplasts harboring a number of FtsZ rings [176]. The *msl1* single mutant plants exhibited phenotype similar to the wild-type plants, while the *msl2msl3* double mutants and *msl1msl2msl3* triple mutant plants exhibited leaf rumpling. The introduction of an *msl1* lesion in the *msl2msl3* mutant suppressed the *msl2msl3* phenotypes. The *msl2msl3* double mutant phenotype showed partial dependence on MSL1 function, indicating inter-organellar communication between plastid and mitochondria [177].

MSL8 acts as a mechanotransducer involved in pollen activity during hydration as well as germination [178]. MSL10 not only acts as an ion channel but also induces phosphorylation-regulated cell death through its plant-specific N-terminal domain. Overexpression of *MSL10* in Arabidopsis resulted in the transcriptional activation of ROS- and cell death-related genes, reduction in stature, H_2_O_2_ generation, and ectopic cell death [179].

MCA genes show broad expression in various tissues [167]. OsMCA1 is located at the plasma membrane and regulates Ca^2+^ influx and ROS production under hypo-osmotic stress. The suspension-cultures overexpressing *OsMCA1* showed an enhanced Ca^2+^ uptake and ROS production while *OsMCA1*-suppressed cells harboring Ca^2+^-sensitive photoprotein aequorin exhibited partially impaired changes in free [Ca^2+^]_cyt_ levels under hypo-osmotic stress. The *OsMCA1*-RNAi transgenic plants exhibited growth retardation and shorter rachises [180]. AtMCA1 and AtMCA2 are involved in Ca^2+^ uptake in roots as well as plant growth. The plasma membrane-localized MCA1 is involved in Ca^2+^ uptake in Arabidopsis roots. The *MCA1-*OE lines showed enhanced Ca^2+^ uptake and the free cytosolic Ca^2+^ levels under hypo-osmotic stress. The primary root of *mca1* seedlings lacked the ability to penetrate harder agar medium [181]. The *mca2* and *mca1mca2* mutant plants exhibited defective Ca^2+^ uptake, with the double mutant plants exhibiting enhanced sensitivity to excess Mg^2+^ levels and slower growth as compared to the wild type plants. The primary root of the *mca2* seedlings is normal in the perception of the hardness of agar [182]. NtMCA1 and NtMCA2 are involved in Ca^2+^ uptake in BY-2 cells and the consequent cell proliferation as well as gene expression under mechanical stress [183]. MCA1, CYTOKININ RECEPTOR1 (CRE1) and RBOHDF-derived ROS regulate osmosensitive metabolic alterations, thus, indicating that MCA1-mediated mechanoreception is involved in cellulose and carbohydrate metabolism [184]. MCA1 and MCA2 are involved in cold tolerance as well as CBF/DREB1 (C-repeat responsive element binding factor/dehydration-responsive element binding factor)-independent cold signaling in Arabidopsis. The *mca1* and *mca2* mutant plants exhibited lower elevations in [Ca^2+^]_cyt_ levels than in wild type plants under cold stress, while the *mca1mca2* double mutant plants exhibited enhanced sensitivity to chilling and freezing. The *mca1mca2* double mutant plants showed downregulated expression of *CBF2-*independent, heat stress transcription factor C-I (*HSFC1*)-dependent expression of cold-inducible genes, *At5g61820* (encoding an unknown protein), *At3g51660* (encoding tautomerase/MIF superfamily protein) and *At4g15490* (encoding UDP-glycosyltransferase superfamily protein UGT84A3) [185].

An Arabidopsis piezo protein, AtPiezo, is an ortholog of its animal counterparts and acts as a mechanosensitive cation channel. It is induced by a viral infection and has the potential as an antiviral mechanism due to the presence of a single piezo ortholog in a number of plant species [169]. OSCA1 is a plasma membrane localized hyper-osmolality-gated Ca^2+^-permeable channel and may be an osmosensor [92]. The Arabidopsis Defective Kernel 1, DEK1 protein, involved in early-embryonic and post-embryonic development, is dependent on mechanically activated Ca^2+^-regulated perception of mechanical stress. The existence of novel mechanisms of mechanosensitive stress perception in plants is a challenge to our understanding [186].

### 3.6 Ca^2+^-ATPases

Ca^2+^-ATPases are high-affinity but low-capacity transporters [96]. The plant P-type Ca^2+^-ATPases belong to the P- type superfamily of ATPases and are of two types: IIA/P2A and IIB/P2B, with the latter containing a CaM binding autoinhibitory domain at the N-terminal that activates the Ca^2+^ pump [187]. The type IIA or ER-type Ca^2+^-ATPases (ECAs) and the type IIB or autoinhibited Ca^2+^-ATPases (ACAs) are localized on the endomembranes as well as the PM [93, 188]. Ca^2+^-ATPases comprise ten transmembrane segments with TM1-6 forming the transport domain, T-domain. The rest of the segments form a support domain, S-domain, that is involved in the coordination of ion binding and supports the T-domain structurally. The cytosolic domains include phosphorylation domain (P), actuator domain (A) and nucleotide-binding domain (N) are involved in ATP hydrolysis. ACA allows Ca^2+^ transport only, while ECA may regulate the transport of divalent cations like Zn^2+^, Cd^2+^ and Mn^2+^ [93, 189].

Arabidopsis genome encodes four ECAs (AtECA1-4) and ten ACAs (AtACA1, 2, 4, 7-13) [190]. The identification of forty-two genes encoding 33 TaACA and 9 TaECA proteins in *T. aestivum* was accomplished through genome-wide identification [191]. Rice genome encodes ten ACAs (OsACA1-10) and two ECAs (OsECA1 and OsECA2) [101]. The soybean genome encodes eight genes belonging to the P2A sub-family and twenty-five genes belonging to P2B subfamily. The Ca^2+^-ATPase family shows little variation in the length of protein sequence in soybeans (752-1103 residues), but show high variability in intron numbers (0-36) [105]. The P2 type ATPases in Arabidopsis, rice, and soybean suggest their common ancestry [192].

Ca^2+^-ATPases regulate a number of developmental and adaptive responses. The *OsACA6* gene identified in *O. sativa,* encodes a type IIB Ca^2+^-ATPase and has been implicated in tolerance to ABA, heat, salt and drought stresses [193]. Microarray analysis of post-pollination stigma and post-adhesion of the pollen coat led to the identification of the *ACA13* gene. ACA13 acts as a Ca^2+^ pump and is involved in pollen germination as well as the growth of pollen tube in Brassicaceae [194]. Similarly, ACA7 has been implicated in pollen development [195]. ACA10 is involved in the regulation of elongation during vegetative growth in adult plants as well as in inflorescence structure in Arabidopsis [196]. ACA8, localized at plasma-membrane, is involved in sucrose signaling during nascent stages of seedling development and co-ordination of root development in Arabidopsis [197]. CrACA1 is reported to be involved in the gravity-perception mechanism in spores of *Ceratopteris richardii.* It is localized in the plasma membrane and is involved in early germination as well as gravity-regulated polarity. Transcriptomic studies indicate its role in Ca^2+^ efflux at the trans-cellular level [198]. The *OsACA7* gene encoding a Ca^2+^-ATPase imparts tolerance to salinity stress in hypersensitive K616 yeast mutant and may play a similar role in plants. It is upregulated during panicle and seed development as well as under abiotic stresses [100].

The P-type cation-transporting ATPase (*PCA1*) gene, identified in *Physcomitrella patens*, encodes a type IIB Ca^2+^-ATPase. It is involved in salinity stress-induced Ca^2+^ signaling pathway [199]. ER-localized *Nicotiana benthamiana* Ca^2+^-transporting ATPase (NbCA1), a type IIB Ca^2+^-ATPase, is involved in Ca^2+^ efflux pathway that regulates programmed cell death (PCD). RNAi silencing of *NbCA1* enhances PCD in the case of N-immune receptor, Cf9 receptor, *Pst* strain (non-host pathogen) and cryptogein. NbCA1 acts upstream of NbCAM1 as well as NbrbohB that act as Ca^2+^ decoders in N-immune receptor-regulated PCD [200]. *ACA4* and *ACA11* act as genetic suppressors of PCD, thus, connecting PCD with vacuolar Ca^2+^ signals in plants. The double knockout Arabidopsis mutants of *ACA4* and *ACA11* develop hypersensitive response-like lesions. The loss of ACA4 and ACA11 vacuolar pumps augments the activation of an SA-dependent PCD in Arabidopsis [201]. ACA4 and ACA11 are involved in Filaggrin or filament aggregating protein 22 (flg22)-dependent Ca^2+^ signaling. The *aca4/11* mutants exhibited enhanced basal Ca^2+^ levels and Ca^2+^ signals on exposure to flg22. The restoration of the defense-related *aca4/11* phenotype occurs through re-localization of ACA8 from the plasma membrane to the tonoplast, indicating that the tonoplast-localized Ca^2+^ flux is critical towards the maintenance of Ca^2+^ homeostasis as well as the host immune responses [202].

ACA8 complexes with leucine-rich repeat receptor kinase (RK) FLAGELLIN SENSING2 (FLS2) *in planta*. ACA8 as well as its closest homolog, ACA10, are involved in the restriction of virulent bacterial growth. The *aca8aca10* knockout mutant plants exhibit a fall in flg22-induced Ca^2+^ levels and ROS bursts as well as alterations in transcriptional reprogramming. The flg22-induced gene expression is enhanced in case of regulation through MAPKs while it is decreased in case of regulation through CDPK. ACA8 interacts with BRI1 and CLV1 receptor-like kinases (RLKs), with the morphological phenotypes of *aca8* and *aca10* mutant plants implicating ACA8 and ACA10 in the regulation of developmental processes [203]. They also interact with the conserved BONZAI1 (BON1) protein and are involved in the Ca^2+^ signaling pertaining to stomatal movement and the consequent immunity. The *aca10* as well as the *bon1* mutants exhibit an autoimmune phenotype and enhanced Ca^2+^ steady levels. BON1 acts as a positive regulator of the ACA10 and ACA8 function [204]. ACA and its isoforms are critical to adaptive responses to various abiotic stresses.

### 3.7 Ca^2+^/H+ Exchangers (CAXs)

CAX transport proteins are members of the multigene family of CAXs [94, 205, 206]. They are low-affinity but, high-capacity transporters [96]. CAXs have been identified in bacteria, fungi, plants, and several animals but maybe absent in archaebacteria, *Caenorhabditis elegans*, insects and mammals. CAXs are categorized as Types I (based on similarity to Arabidopsis CAX1), II (based on similarity to *S. cerevisiae* VNX1) and III (based on similarity to *E. coli* ChaA) [97, 207]. Arabidopsis genome harbors six *CAX* genes [97], while other plant genomes may harbor 4-14 *CAX* genes [94, 97]. Rice genome encodes sixteen Ca^2+^ exchangers that include eight CAXs and four CCXs [100]. The soybean genome contains eighteen *CAX* genes and eight *CCX* genes [105]. The Ca^2+^/cation antiporters include AtCAX7-AtCAX11 transporters in Arabidopsis. They encode K^+^-dependent Na^+^/Ca^2+^ exchangers and regulate active cytosolic Ca^2+^ efflux [97, 207]. CAXs contain 11 transmembrane helices and form either homomeric or heteromeric oligomers [94]. The transmembrane regions have highly conserved primary amino acid sequences in plant CAXs, and the loop and tail regions account for most of the sequence diversity [97]. CAXs possess an autoinhibitory domain at the N-terminal and a significantly conserved cation-binding region. The nine-amino-acid region between transmembrane domains TM1 and TM2 determines their transporter specificity [97, 205]. CAX activity is influenced by phosphorylation, alterations in pH, regulatory proteins like the SOS2 (Salt Overly Sensitive) kinase and stresses [208].

The *cax1* mutant plants showed a decrease in tonoplast Ca^2+^/H^+^ antiporter activity and tonoplast V-type H^+^-translocating ATPase activity as well as an enhanced tonoplast Ca^2+^-ATPase activity and expression of *CAX3* and *CAX4*. The mutant plants exhibited increased growth under Mn^2+^ and Mg^2+^ stress. Also, they showed alterations in development, hormone sensitivities and stress responses [209]. The *cax3* mutants exhibited modest sensitivity to exogenous Ca^2+^ as well as a decrease in the activity of vacuolar H^+^-ATPase. The *cax1cax3* mutant plants exhibited reduced growth, ionic sensitivity, leaf tip as well as floral necrosis, with the partial suppression of these defects through the application of exogenous Mg^2+^. They also showed a reduction of H^+^-ATPase activity and vacuolar H^+^/Ca^2+^ transport [210].

CAXs are involved in the regulation of apoplastic pH [211, 212]. CAX1 and 3 show highly cell-specific function in the organellar distribution of Ca^2+^ [213]. CAX1, ACA4 and ACA11, located in tonoplast, show preferential expression in Ca^2+^-rich mesophyll [211]. The *cax1* and *cax3* mutant plants showed enhanced sensitivity to ABA as well as sugar during the germination phase, enhanced tolerance to ethylene in the early stages of seedling development. The *cax3* plants exhibited sensitivity to lithium, salinity as well as low pH levels due to a decrease in vacuolar H^+^/Ca^2+^ flux under salinity stress as well as activity of plasma membrane H^+^-ATPase [214]. *CAX1* and *CAX3* are expressed in guard cells. They show co-expression in mesophyll on exposure to wounding or flg22. The formation of a functional CAX1- CAX3 complex and the resulting interactions between the two are involved in stomatal functioning [215]. CAX11 is involved in the maintenance of cytosolic Ca^2+^ signaling under hypoxic stress in root cells [216]. The CCHA1 (a chloroplast-localized potential Ca^2+^/H^+^ antiporter) identified in Arabidopsis may be involved in the regulation of photosystem II (PSII) as well as Ca^2+^ and pH homeostasis [217]. The *cax1* mutants have been confirmed to be the only genotype that exhibits a partial degree of tolerance to the magnesium sulfate toxicity comparable to the regolith profile [218]. The *cax1* mutants exhibited tolerance to serpentine soils as well [219].

### 3.8 Cation/Ca^2+^ Exchangers (CCXs)

The cation/Ca^2+^ exchanger (CCX) sub-family in Arabidopsis comprises five members, CCX1-CCX5, earlier classified as belonging to the CAX sub-family (CAX7-CAX11) [207, 220]. Arabidopsis *CCX1* is highly induced during leaf senescence. CCX1 may be involved in the regulation of leaf senescence through ROS homeostasis and Ca^2+^ signaling as the *ccx1-1* and *ccx1ccx4* plants showed more sensitivity to Ca^2+^ deficient conditions. The *ccx1ccx4* mutant plants displayed a stay-green phenotype under natural as well as dark-induced leaf senescence and on exposure to H_2_O_2_. The *CCX1*-OE lines showed ROS accumulation and enhanced senescence [221]. Arabidopsis CCX2 localizes to the ER and shows strong induction under salinity and osmotic stresses. The Arabidopsis *ccx2* mutant plants showed less tolerance to osmotic stress and reduced root/shoot growth under salinity stress as well as reduced cytosolic and enhanced ER Ca^2+^ levels in both stresses [222]. AtCCX3, an endomembrane-localized H^+^-dependent K^+^ transporter, is involved in K^+^, Na^+^ and Mn^2+^ transportation. *AtCCX3* expression is observed in flowers, whereas *AtCCX4* is broadly expressed throughout the plant. The *ccx3* and *ccx4* mutant lines show no visible growth defects while *AtCCX3*-OE showed high Na^+^ levels. Tobacco plants overexpressing *AtCCX3* resulted in lesions in the leaves, stunting, and higher cationic levels [223]. AtCCX5, localized at the plasma membrane and nuclear periphery, regulates high affinity K^+^ uptake and Na^+^ transport in yeast [224]. Of the four CCXs in rice, *OsCCX2* transcripts show differential expression under abiotic stresses and down-regulation under Ca^2+^ deficient conditions [225]. As we have discussed under each sub-section of this section, it is evident that the role of each component is critical to plant development as well as stress regulation. However, we still need further investigations into their functional aspects, particularly their mechanisms, to be able to utilize their complete potential. Calcium transport machinery needs further attention in plant systems owing to their involvement in pathological conditions in animal systems. Their physiological functions under various stresses will help us understand and utilize their potential significantly in the changing climatic conditions.

## 4 RESPONSE AND ADAPTATION UNDER STRESS

Plant’s growth and development is greatly affected by environmental stress. Improvement in survivability and growth is a major subject in crop selection and breeding under stress conditions. Plants have developed specific signal transduction pathways to respond to specific stresses and thus, adapting themselves to unfavorable conditions. Here, we describe the role of Ca^2+^ signaling components involved in mediating ABA signaling, ion homeostasis, biotic and abiotic stresses responses.

### 4.1 ABA Signaling

ABA plays an essential role in many developmental and physiological events such as seed maturation, dormancy, stomatal regulation and abiotic stress responses [226-228]. The role of ABA in stomatal regulation is a well-known phenomenon. Besides ABA, various other factors, including CO_2_, ozone, humidity, light, pathogens *etc.* are also involved in the regulation of stomatal movements [229]. ABA inhibits the inward K^+^ channel and hence, prevents the assimilation of K^+^ in the guard cell. Furthermore, it stimulates anion channels to reduce the guard cell turgor pressure thereby, promoting stomatal closing [229-232].

The involvement of Ca^2+^ elements in mediating stomatal movements and the cross-talk between Ca^2+^ and ABA signaling is vital in the study of stomatal opening and closure under drought stress conditions. The opening and closing of stomata depend on transient changes in [Ca^2+^]_cyt,_ which is promoted by various Ca^2+^ sensors [233-235]. Regulation of stomatal closure is accomplished by more accumulation of anions in the cytoplasm, mediated by anion channels in guard cell. The activation of anion channels is, directly and indirectly, dependent on Ca^2+^ and ABA, respectively. SLAC1 (Slowly anion channels associated-1) generates S- type anion currents in the guard cell [236, 237]. It is activated by a Ca^2+^-independent protein kinase *i.e.,* sucrose non-fermenting-1 (SNF-1)-related serine/threonine-protein kinase-2 (SnRK2) as well as Ca^2+^-dependent PKs such as CDPK6, CDPK21 and CDPK23. However, these kinases are inactivated by PP2Cs such as ABSCISIC ACID INSENSITIVE 1 (ABI1), ABI2, or PROTEIN PHOSPHATASE 2CA (PP2CA) [238-241]. Whereas, in the presence of ABA, pyrabactin resistance (PYR)/PYR-like (PYL), cytosolic ABA receptors further inactivate PP2Cs activity [242, 243]. The kinases and phosphatases are essential components of Ca^2+^ signaling events, which balance the overall signal transduction mechanism to promote plant growth under stress.

Several CDPKs and CIPKs have been reported to be involved in the regulation of ABA signaling. CDPK8 is an essential inducer of tetrameric iron porphyrin protein, CATALASE3 (CAT3), which participates in ABA-dependent stomatal movement under drought stress. Hence, CDPK8 is a positive regulator of ABA-dependent stomatal movement [244-246]. However, CDPK9 is involved in the negative regulation of ABA-mediated stomatal movements [232]. Additionally, CIPK6 participates in ABA signaling, drought and salinity stress responses in *Arabidopsis* [247]. The involvement of *CIPK* genes in ABA signaling and abiotic stresses has also been observed in foxtail millet [78].

Plant CBLs and CMLs have also been involved in the ABA signaling pathway during developmental programming and stress responses. CBL1 and CBL9 interact with CIPK23 and together, the CBL-CIPK complex regulates ABA mediated stomatal responses and also low K^+^ uptake in Arabidopsis [248]. The CBL9-CIPK3- ABA repressor 1 (ABR1) pathway regulates ABA responses during seed germination as well as the adult developmental stages [249]. AtCML9 has a potential role in abiotic stress responses induced by ABA. The loss-of-function *atcml9* mutants showed hypersensitive responses with more tolerance to salt and drought stress [250]. However, AtCML20 is a negative regulator of ABA-induced stomatal movements. The *atcml20* mutant lines showed hypersensitivity towards ABA-inhibited K^+^_in_ channels and ABA-promoted S-type anion channels in comparison to control plants [251].

Ca^2+^ pumps and channels are responsible for the generation of Ca^2+^ fluxes to mediate Ca^2+^-dependent ABA signaling in plants. The involvement of plasma membrane-localized Ca^2+^-ATPases and the vacuolar channel activity of TPC have been reported in ABA signaling and its effects on ABA-induced stomatal regulation [138, 252].

### 4.2 Ionic Homeostasis

The regulation of ion homeostasis is important to protect the cell from ion toxicity. Ca^2+^ signaling is associated with ion homeostasis in plants. Magnesium (Mg), potassium (K) and nitrogen (N) are primary macronutrients required for various cellular processes. Whole-genome transcriptomic studies have revealed that short and long term Ca^2+^ starvation responses in rice result in differential gene expression, with the longer terms inducing larger variation. Transcriptomic data in case of nutrient deficiencies (N, P, K) in rice and Arabidopsis showed similar responses of differentially expressed genes. The K^+^-responsive genes are involved in metabolism, stress responses, signal transduction, transcriptional regulation, ion transport and those related to growth and development. Several genes responsive to low-K^+^ conditions show a reversal in expression upon restoration of K^+^ levels [253, 254]. The genes showing transcriptional alterations under low-K^+^ stress showed similar patterns in rice and Arabidopsis, although more stress-responsive genes and development-related genes were influenced in Arabidopsis [255].

A number of studies have identified the role of CBL/CIPK complexes in the activation and deactivation of K^+^ transporters under low K^+^ stress. *CIPK9* is involved in low-K^+^ responses [256]. AP2C1, a PP2C, interacts with and dephosphorylates CIPK9 in cytoplasm, which was confirmed by bimolecular fluorescence complementation (BiFC), fluorescence resonance energy transfer (FRET) and co-localization studies. The *ap2c1* mutant lines were tolerant, whereas the transgenic over-expression lines were sensitive under low K^+^ conditions [257]. However, *cipk9* mutant lines are sensitive in low K^+^ conditions [256]. Thus, CIPK9 acts as a positive regulator and AP2C1 acts as a negative regulator in the root growth of *Arabidopsis* under low K^+^ conditions [257]. CIPK23 is also involved in low K^+^ stress response in Arabidopsis. During K^+^ deficiency, CBL1 and CBL9 target CIPK23 to the plasma membrane of the root and the latter phosphorylates *Arabidopsis* K^+^ transporter1 (AKT1), an inwardly rectifying K^+^ channel, thus, mediating the K^+^ uptake in the cell [248, 258-260]. A group of CIPKs including CIPK23, CIPK16 and CIPK6 interact with multiple CBLs to phosphorylate and activate AKT1 [74, 241, 260, 261]. Interestingly, a PP2C-type phosphatase, AIP1, dephosphorylates CIPK23 and act as a negative regulator in the pathway of CIPK23-dependent AKT1 activation [74]. Different CIPKs interact with different phosphatases to regulate the activity of AKT1. CIPK23 and CIPK16 interact with AIP1 and AIP1H (a PP2C-type phosphatase), CIPK6 interacts with four different PP2Cs, AIP1, AIP1H, AHG1, and PP2CA [8, 261]. The CBL-CIPK-PP2C pathway is responsible for the regulation of K^+^ homeostasis in plants. CIPK23 regulates high-affinity K^+^ transporter 5 (HAK5) in Arabidopsis [262]. AtCIPK1 and AtCIPK9, along with AtCBL1 or AtCBL9, are involved in the regulation of high-affinity K^+^ uptake through AtHAK5 [263]. AtCIPK23 and Shaker α-subunit, AtKC1, act synergistically to modulate AKT1-mediated low K^+^ responses [264]. CBL10 interacts with AKT1 directly and is involved in the negative regulation of AKT1 activity [265].

In addition to K^+^ homeostasis, the CBL-CIPK signaling network plays a significant role in the regulation of Mg^2+^ homeostasis. The *cbl2cbl3* double mutants were highly sensitive in excessive Mg^2+^ conditions. Further on, with the help of ionic profile analysis, these lines showed reduced Mg^2+^ accumulation in the cytoplasm, indicating that CBL2/CBL3 are involved in maintaining Mg^2+^ concentration in higher plants. CIPK3/CIPK9/CIPK23/CIPK26 interact with CBL2/CBL3 and regulate Mg^2+^ homeostasis by sequestering excess Mg^2+^ into the vacuole [266, 267].

Nitrate (NO_3_^-^) is a principal source of nitrogen (N), required for crop production and yield [268]. In *Arabidopsis,* three members (AtNRT1, AtNRT2 and AtCLC) of the nitrate transporter family have been identified [269-271]. AtNRT1 and AtNRT2 are both involved in the uptake of NO_3_^-^ in the root cell [269-272]. Arabidopsis AtNRT1.1 (CHL1) is a dual-affinity nitrate transporter. Phosphorylation at Thr residue 101 results in its functioning as a high-affinity NO_3_^-^ transporter whereas its dephosphorylation makes it a low-affinity NO_3_^-^ transporter [273, 274]. It participates in nitrate signaling *via* regulation of *AtNRT2.1* expression [275]. The role of Ca^2+^ in nitrate signaling is demonstrated by the involvement of CBL-CIPK interactions. *AtCIPK23* was downregulated in the *chl1* mutants in microarray analysis. Moreover, AtCIPK23 phosphorylates AtNRT1.1 at its Thr 101 position. Thus, AtCIPK23 is a key regulator for the transport and signaling of AtNRT1.1. CIPK8 acts as a positive regulator of the low-affinity nitrate responses, whereas CIPK23 acts as a negative regulator of the high-affinity nitrate responses [273]. CIPK23 also phosphorylates a high-affinity ammonium (NH_4_^+^) transporter, AMT1, at its Thr residue located at the cytosolic C-terminus and deactivates its transport activity, thus, protecting plants from the toxic NH_4_^+^ levels [276].

Phosphorous (P) is an essential mineral element that participates in a few enzymatic and metabolic pathways in the plant. The upregulated expression of *BnCBL1* and *BnCIPK6* as well as their interaction with each other, implicate them in inorganic P (Pi) deficiency in *B. napus* [277, 278]. A schematic representation of ionic signaling has been depicted in Fig. (**2**).

### 4.3 Biotic and Abiotic Stress Responses

Calcium signaling components have been reported to be involved in plant responses to various biotic and abiotic stresses. The complexity of CaM/CML family may be a determinant of their stress-related responses to diverse stimuli [24]. Silencing of *CaM2* and *CaM6* genes showed an impaired resistance to tobacco rattle virus and the soil-borne phytopathogen, *Pythium aphanidermatum*, indicating that they may be involved in disease resistance of tomato to pathogens [27]. CMLs are involved in the development and stress responses in plants [3, 279]. Arabidopsis CML8 has been identified as a positive regulator of defense responses against *P. syringae* [280]. AtCML9 acts as both a positive regulator of immune responses and a negative regulator of drought stress tolerance in Arabidopsis [250, 281]. It shows early induction in plants on exposure to salinity, cold, drought, bacterial pathogens, ABA and Salicylic acid (SA). It also interacts with the WRKY53 and TGA3 transcription factors [282]. AtCML37 and AtCML42 are involved in the regulation of abiotic stress responses and defense responses against herbivorous insects [283-285]. Arabidopsis CML37 acts as a positive regulator of Ca^2+^ signaling induced by wounding and infestation by *Spodoptera littoralis* [284]. The loss-of-function *AtCML37* mutants showed increased susceptibility to herbivory, coherent with lower expression of JA-responsive genes as well as levels of the bioactive form of Jasmonic acid (JA-Ile) [284]. The *Atcml37* null mutants exhibited a reduced tolerance to drought stress, consistent with low ABA levels [285]. AtCML42 negatively regulates herbivore resistance by downregulating the expression of JA-responsive genes and elevation of levels of defensive secondary metabolites [283]. The *SpCaM* and *SpCML* genes are involved in the regulation of plant responses under biotic and abiotic stresses in *S. pennellii* [28]. The expression profiles of *VviCaM* and *VviCML* genes showed broad expression across tissues during various developmental stages and a significant modulation in biotic stress-related responses [24]. Some *VaCaM* and *VaCML* genes in wild grape (*V. amurensis*) are involved in the positive regulation of abiotic stress tolerance while some *VaCML* genes exhibit negative regulation [286]. Apple (*Malus domestica*) genome harbors four *MdCaM* and fifty-eight *MdCML* genes, which are expressed in multiple tissues as well as in responses to biotic and abiotic stress. The *MdCML3*-OE apple calli showed enhanced tolerance to salinity as well as ABA [287].

The roles of CMLs need more investigations to understand their involvement in biotic and abiotic signaling pathways. The identification of the targets of CaM/CML and their functions is an ongoing pursuit and a limitation to our understanding at present.

The SOS pathway is directly related to salt stress tolerance by regulating Na^+^ homeostasis in plants [288-292]. Three sodium (Na^+^) transporters: Na^+^/K^+^ symporters, Na^+^/H^+^ exchangers in the tonoplast and Na^+^/H^+^ antiporters in the plasma membrane are present in plants [293]. Excess Na^+^ can result in salinity stress in plants. Plants may overcome salinity stress through the accumulation of excessive Na^+^ into vacuole, transport of Na^+^ into soil and Na^+^ recycling in the old tissue [9]. Under salinity stress, AtCBL4/SOS3-AtCIPK24/SOS2 gets activated and this complex regulates the action of Na^+^/H^+^ exchangers to reduce the Na^+^ level in the cytoplasm to protect the plants [294]. Comparative transcriptomic analysis of the leaves of salt-tolerant and -sensitive cotton genotypes showed differential expression of salt tolerance-related genes and miRNAs as well as their weak expression in the salt-sensitive genotype under salinity stress [295]. The *GhCIPK6a-*OE cotton lines showed a significant increase in salt tolerance, with the transgenic cotton exhibiting better growth as well as increased lint content and uniformity of fiber length than wild-type plants in saline field conditions [296]. The *GhCIPK6-*OE lines showed reduced oil content and alterations in the composition of fatty acids [81]. The Tibetan plateau wild barley (*Hordeum spontaneum*) genome harbors twelve *HsCIPK* genes. The overexpression of *HsCIPKs* in rice showed increased tolerance of roots to heavy metal toxicities, salinity and drought stresses [297]. *NtCIPK9* gene has been identified in the halophyte, *Nitraria tangutorum.* The Arabidopsis *NtCIPK9-*OE lines show enhanced salt tolerance and decreased damage due to salt stress by enhancing the expression of genes regulating ion homeostasis [298]. CIPK23 is involved in salt and drought tolerance regulated by Sensitive to Proton Rhizotoxicity1 (STOP1) in Arabidopsis [299].

*CDPK* genes in wheat are involved in responses to gibberellin (GA), ABA, drought, cold and salinity stresses as well as biotic stress responses [47]. The expression and co- expression profiling, as well as prediction of protein-protein interaction network, implicate most of the *BdCDPK* genes in hormonal signaling pathways and physiological responses to stress signals in *B. distachyon*. BdCDPKs might be involved in the activation as well as repression of WRKY- or mitogen-activated protein kinase (MAPK)-regulated stress responses [49]. The sustained activation of CDPK4/5/6/11 phosphorylates WRKY transcription factors such as WRKY8/28/48 directly to regulate transcriptional reprogramming critical for NLR-dependent pathogen restriction in Arabidopsis. Also, CDPK1/2/4/11 phosphorylate plasma membrane-localized NADPH oxidases for ROS generation [300]. The *CDPK* genes of wild grape show differential organ-specific expression. The expression of *VaCPK1*, *VaCPK2*, *VaCPK3*, *VaCPK9*, *VaCPK13*, *VaCPK16*, *VaCPK20*, *VaCPK21*, *VaCPK26* and *VaCPK29* genes is induced by abiotic stress implicating them in response to temperature and osmotic stresses [301]. Genome-wide analysis of *Glycyrrhiza uralensis*, an important medicinal plant, led to the identification of twenty-three *GuCDPK* genes. These genes may be involved in the synthesis of glycyrrhizic acid and flavonoids under salt stress [302]. The cassava genome also harbors twenty-seven CDPKs, that are involved in responses to abiotic stress [303]. The expression profiles of seventeen *CDPK* genes in pineapple in response to different stresses, implicate them in a number of signaling pathways [304]. Transcriptomic data of pear seedlings under salt stress showed the activation of genes including those involved in Ca^2+^ signaling [305]. Genomic and transcriptomic analysis of strawberry (*Fragaria ananassa*) led to the identification of nine novel *CDPK* genes. The *FaCDPK* genes show differential expression during fruit development, ripening and responses to abiotic stress [54]. Similarly, forty-four CDPKs have been identified in bananas. Transcriptomic analyses showed enhanced *CDPK* transcript levels during the early fruit development as well as post-harvest ripening. The transcriptional regulation of CDPKs during ripening, development and response to abiotic stress holds potential for genetic improvement [306].

CBL-CIPK interactions play a significant role in the regulation of drought and osmotic stress responses. Expression analysis revealed the upregulation of *TaCIPK2* by ABA, H_2_O_2_ treatment and polyethylene glycol (PEG) in wheat leaf [307]. Additionally, *TaCIPK2* interacted with *TaCBL1*, *2*, *3* and *4* in yeast two-hybrid assays. The over-expression lines of *TaCIPK2* were more tolerant to drought and osmotic stress conditions with green cotyledons and higher survival rates as compared to controls. Physiological index analyses revealed that transgenic lines showed low water loss rate and ion-leakage with less accumulation of malondialdehyde, H_2_O_2,_ and higher activity of SOD and CAT as compared to control plants. Additionally, the stomatal closure was much faster in transgenic lines under osmotic stress treatment [307].

Several reports suggest the involvement of CBL-CIPK interaction under cold stress conditions. The CBL1-CIPK7 complex may be involved in cold stress responses of *Arabidopsis* [308]. Eight CBLs and twenty-five CIPKs have been identified in tea plant (*Camellia sinensis* (L.) O. Kuntze). Expression analyses indicate their developmental stage specific interaction and involvement in cold stress responses. CsCBL1 interacted with CsCIPK1/CsCIPK10b/CsCIPK12 and CsCBL9 with CsCIPK1/CsCIPK 10b/CsCIPK12/CsCIPK14b in yeast two-hybrid assays. *CsCIPK4*/ *CsCIPK6a*/*CsCIPK6b*/*CsCIPK7*/*CsCIPK11*/*CsCIPK14b*/*CsCIPK19*/*CsCIPK20* and *CsCBL9* were upregulated both in young shoots and mature leaves under cold stress conditions. However, *CsCBL1*/*CsCBL3*/*CsCBL5* and *CsCIPK1*/*CsCIPK8*/*CsCIPK10a*/*CsCIPK10b*/*CsCIPK10c*/*CsCIPK12*/*CsCIPK14a*/*CsCIPK23a*/*CsCIPK24* were induced only in mature leaves, whereas *CsCIPK5*/*CsCIPK25* are induced in young shoots [309].

*CBL* and *CIPK* genes are induced during tissue development, salinity, drought, high and low-temperature stresses in cassava [79]. Similarly, CBL-CIPK interactions are reported to regulate cold, NaCl and wound stress responses in pea (*Pisum sativum*) [310]. The eight CBLs and twenty-one CIPKs in pineapple show differential expression in various tissues. The ectopic expression of *AcCBL1* in *Arabidopsis* renders the plants more tolerant to osmotic stress, salinity stress and fungal infection [311]. The CBLs and CIPKs are more involved in responses to abiotic stress than developmental pathways in grapevine. VvCBLs are particularly involved in the regulation of low temperature responses [72]. Comparative analyses of the CBL-CIPK networking in canola, Arabidopsis and rice have shown functional variations [73].

CBL-CIPK interactions play an essential role in biotic stress responses as well. Nine CaCBLs and twenty-six CaCIPKs have been identified in pepper (*Capcicum annum* L.); almost all were induced under stress conditions. CIPK1 is involved in biotic stress responses in *C. annum*. The ca*cipk1* mutant lines are sensitive to the fungal pathogen, *Phytophthora capsici*. The *CaCIPK1-*OE lines showed enhanced gene expression for defense activity with more H_2_O_2_ accumulation and cell death [312]. The upregulated expression of *TaCBL4* in wheat (*Triticum aestivum*) on infection with *Puccinia striiformis* f. sp. *tritici* and increased susceptibility in the loss-of-*TaCBL4* function mutants, implicate TaCBL4 in defense responses. TaCBL4 interacts with TaCIPK5 implicating them in the positive regulation of defense mechanisms against fungal infection in wheat [313]. Together these reports signify the important role of Ca^2+^ signaling components in abiotic and biotic stress responses. This regulation of stress responses may be targeted in the development of stress-tolerant crops.

## 5 Ca^2+^ SIGNALING MACHINERY: POTENTIAL FOR CROP IMPROVEMENT

Ca^2+^ is an important second messenger that is involved in plant responses to various stress factors and developmental cues. Ca^2+^ sensors perceive changes in cytoplasmic Ca^2+^ levels triggered by different stimuli and set off a systematic chain of events that result in related gene expression. The interaction of signaling elements involves critical molecular targets that can be regulated through genetic manipulation to enable crop improvement. The elements involved in Ca^2+^ signaling machinery are involved in developmental pathways as well as stress responses in plants. The use of the functional genomics approach has led to the identification of important components in Ca^2+^ regulated signaling pathways. Their elucidation and genetic manipulation will aid the development of biofortified fruits as well as enhance stress tolerance in plants. The molecular mechanisms regulating Ca^2+^ signaling pathways under stress and the improvement of traits through molecular breeding of crop plants hold the key to the development of stress-tolerant crops. The CDPKs, CBL-CIPK interactions and transport elements in crop plants will help frame strategies for developing stress tolerance. The *in planta* characterization of various transporters and their genetically engineered variants can help explore more candidates under several stresses. A schematic representation showing the role of Ca^2+^ signaling components in crop improvement is presented in Fig. (**3**).

Most observations have been reported in Arabidopsis and other species, but their functional analyses in crops is limited [100]. Soybean genome contains about 2.0 and 2.3 times greater Ca^2+^ transport protein-encoding genes as compared to Arabidopsis and rice genome, respectively. Arabidopsis genome is reported to contain 104 putative genes encoding Ca^2+^ transport proteins and rice genome contains 89 genes. The soybean genome contains 207 genes encoding putative Ca^2+^ transporters [105]. This indicates that the identification and functional analysis of Ca^2+^ transporters in different crop plants will therefore be an important step towards their genetic manipulation. A significant number of the genes encoding Ca^2+^ transport elements have been shown to be involved in stress responses as well as critical developmental stages, for instance, the panicle and seed development stages in rice [100]. Functional characterization of Ca^2+^ transport elements in rice, including OsCCX2 and other crops will help in the development of stress tolerance, biofortification through high Ca^2+^ levels to address Ca^2+^ malnutrition as well as phytoremediation. The *CAX4*-expressing tomato plants showed increased Ca^2+^ levels and shelf life indicating that CAX expression may help in Ca^2+^ fortification [314]. The over-expression of *AtsCAX1* in potatoes resulted in up to three-fold increase in Ca^2+^ levels [315]. Tomato plants expressing Arabidopsis *sCAX2A* showed enhanced Ca^2+^ levels [316]. Transcriptomic data of high Ca^2+^ finger millet genotype, GPHCPB45, exhibited higher expression of Ca^2+^ transporters (*CAX-1*, *CAX-3*), *CIPK-24* and *CaM* [317]. A node- expressed transporter gene, *OsCCX2*, encodes a putative cation/Ca^2+^ exchanger. OsCCX2, localized to the plasma membrane, is expressed in the xylem of vascular tissues at the nodal regions, thus, loading Cd into xylem vessels. The *osccx2* knockout plants generated by CRISPR/cas9 editing showed a significant decrease in Cd content as well as root-to-shoot Cd translocation ratio [318]. OsCCX2 shows distinct transportation of monovalent and divalent cations in yeast cells. It can be exploited to address phytoremediation in case of soils having high levels of heavy metals [225]. The Arabidopsis *CAXcd*-expression in petunia plants showed higher Cd tolerance and accumulation, without affecting plant development until the flowering stage and seed maturation [319].

## CONCLUSION

The future line of research requires focusing on cross-talk among different pathways as well as the synergistic and antagonistic interactions involved therein. The identification of genes and gene families encoding a number of Ca^2+^ sensors and transporters as well as their working in different scenarios and crop systems, offers possibilities to manipulate plant responses under stress. In addition to genetic approaches, the use of biochemical, microscopic and electrophysiological approaches has also significantly contributed to our understanding of Ca^2+^ influx and efflux. Together, the delineation of signaling pathways has been a holistic approach that has paved the way of genetic manipulation. The identification of different signaling modules operating under different environmental stresses has remarkably opened up avenues for further validations. The progress towards understanding the Ca^2+^ signaling process in a number of plant species has been significant, but the utilization of this knowledge in the context of complexity, specificity or redundancy between signaling components *in planta* is a huge challenge. In addition to that, functioning under different stress responses is a complex process and needs much more scrutiny in the wake of global climatic changes [320-456].

## Figures and Tables

**Fig. (1):**
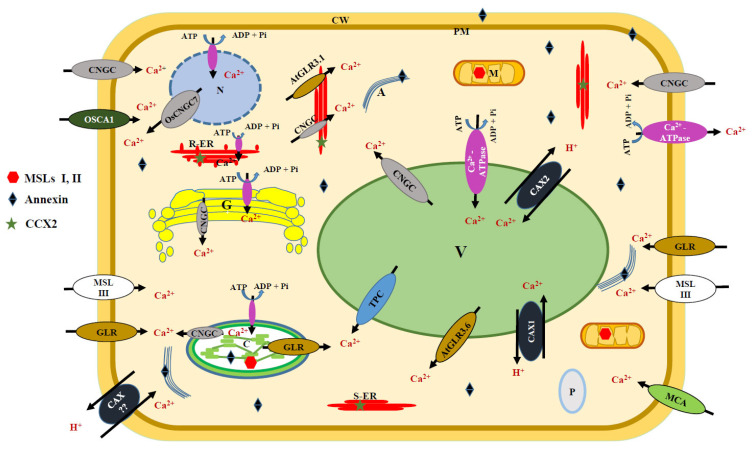
**Subcellular localization of plant Ca^2+^ channels and transporters.** CNGCs, OSCA1, MCA, GLRs, CAX, Ca^2+^-ATPases and Mechanosensitive-like channels (MSLs) type III are located on PM; Ca^2+^-ATPase and CNGC are located on nuclear envelope and golgi membrane; chloroplast carries CNGC, GLR, Ca^2+^-ATPase , annexin (shown as black diamond) and MSLs; GLR3.1, CNGC and CCX2 (shown as green star) reside on ER membrane; Tonoplast carries CNGC, GLR, Ca^2+^-ATPase, TPC and CAX; mitochondria carries MSLs (shown as red hexagon); Annexins are also located on CW, PM, cytosol and actin filaments. Localization is shown on the basis of factors like tissue specificity and species type. CW -cell wall; PM -plasma membrane; N -nucleus; G -Golgi apparatus; C -chloroplast; R-ER -rough endoplasmic reticulum; S-ER –smooth endoplasmic reticulum; V -vacuole; P -peroxisome; A -actin. (Adapted from references [83,92,93,100,222,320] ). (*A higher resolution / colour version of this figure is available in the electronic copy of the article*).

**Fig. (2):**
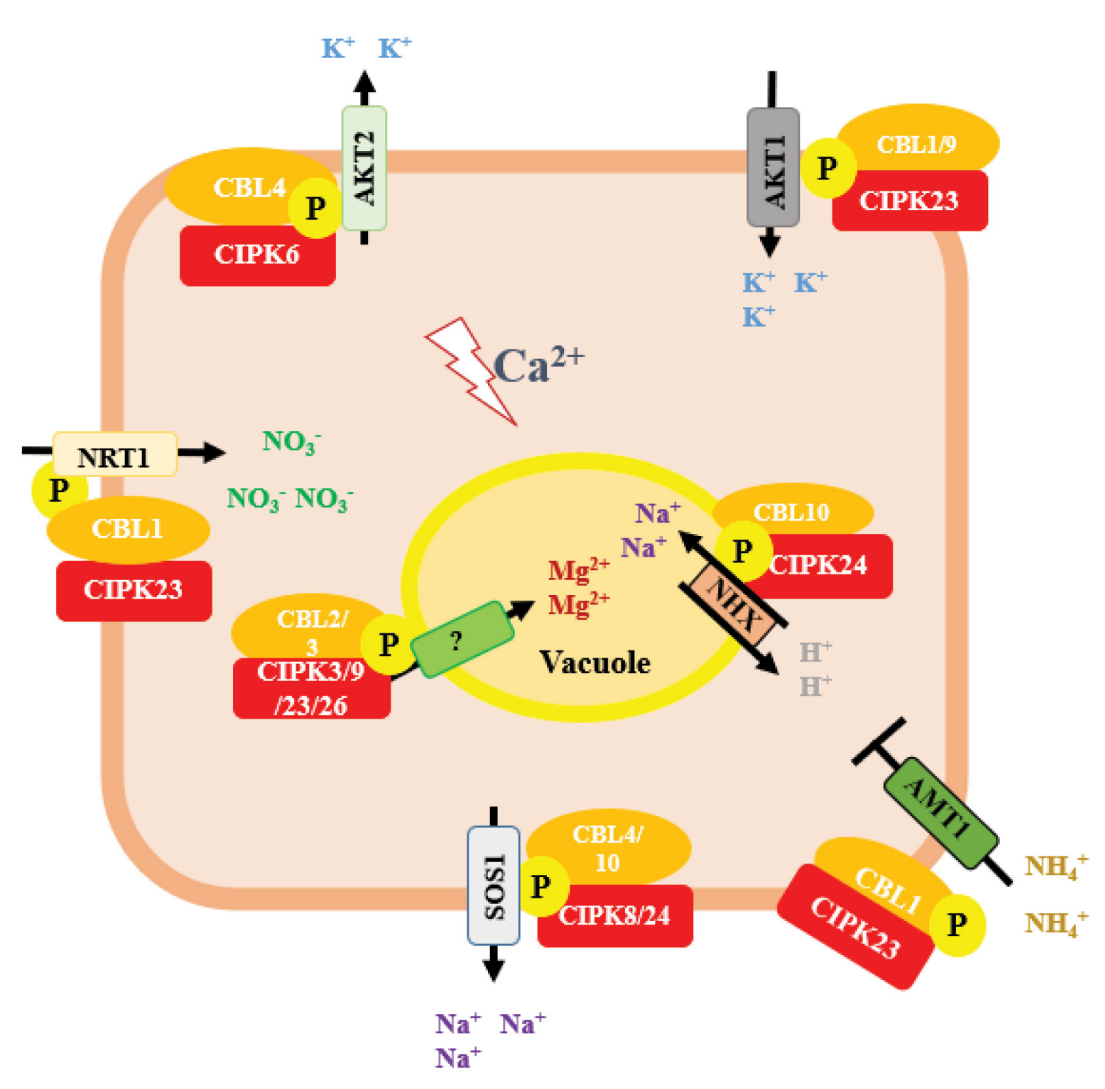
**Regulation of ion homeostasis *via* Ca^2+^ mediated signaling.** Plant CBL-CIPK interactive complexes participate in signal transduction mechanism to regulate ion homeostasis in the cell. CBL1/9 interacts with CIPK23 which phosphorylates AKT1, an inward-rectifying K^+^-channel and mediates K^+^ uptake in the cell. CBL4/CIPK6 interaction is required for AKT2 channel activity and extruding K^+^ from the cell. High affinity NH_4_^+^ transporter 1 (AMT1) is deactivated upon phosphorylation by CBL1-CIPK23 and inhibits NH_4_^+^ uptake in the cell. CBL4/10-CIPK8/24 regulate the activity of Na^+^/H^+^ antiporter (SOS1) to maintain the pH of cell and to reduce salt stress by extruding excessive Na^+^ from the cytoplasm. CIPK23 phosphorylates NRT1, a nitrate transporter and mediate NO_3_^-^ signaling. CBL10-CIPK24 phosphorylate Na^+^/H^+^ exchanger (NHX) localized on tonoplast and maintain Na^+^ level in cell to protect plant from salt stress. CBL2/3 interacts with CIPK3/9/23/26, phosphorylates an unknown Mg^2+^ transporter on tonoplast and mediate Mg^2+^ homeostasis in plant (Adapted from references [266, 276, 321, 322]) (*A higher resolution / colour version of this figure is available in the electronic copy of the article*).

**Fig. (3):**
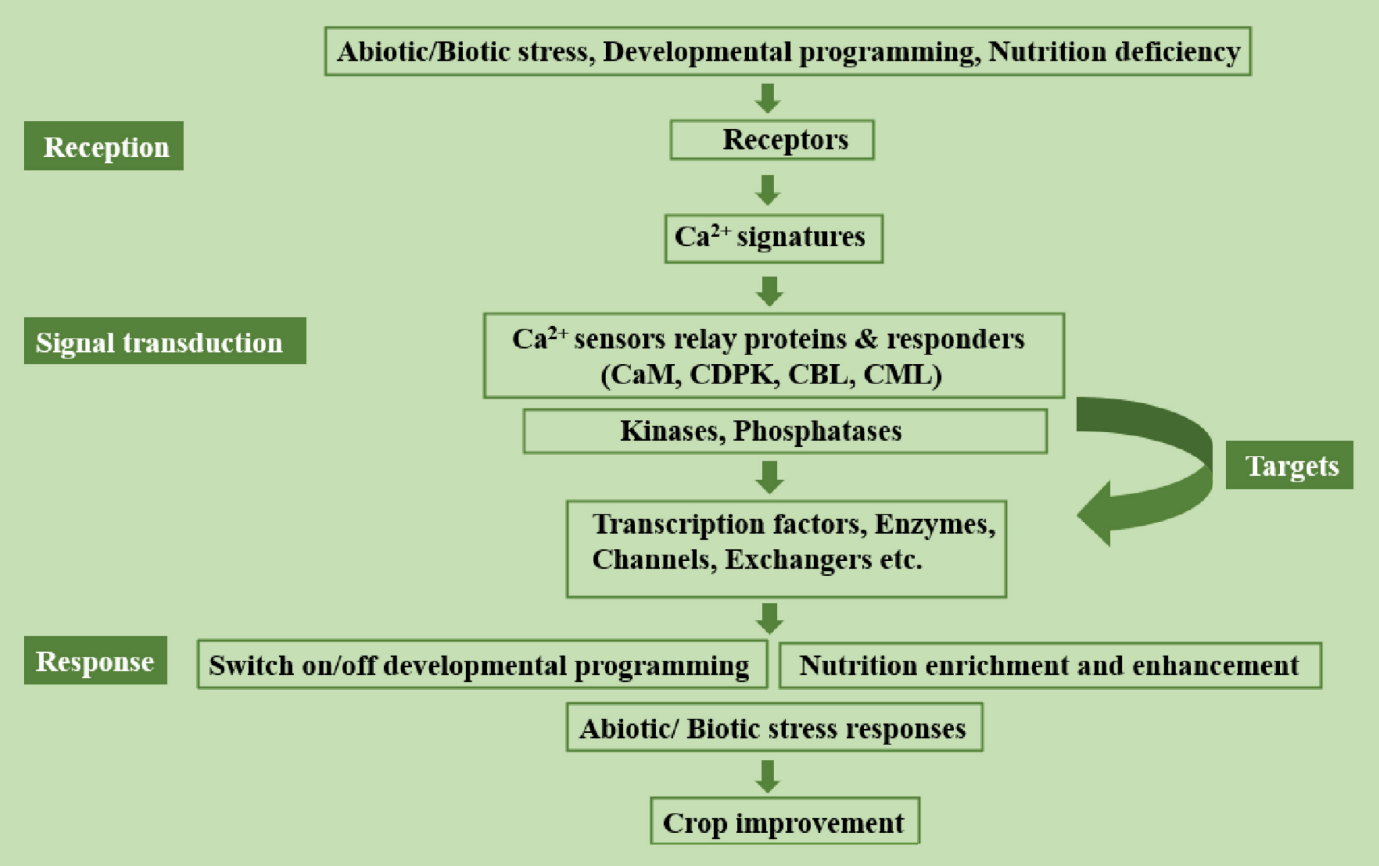
**A schematic diagram showing the role of Ca^2+^ signaling components in crop improvement.** Plants have receptors to sense the stimulus for processing signal transduction mechanism in the cell. A specific Ca^2+^ signature generates in the cytosol for a specific stimulus, responsible for regulating downstream signal cascade. With the help of EF-hands, Ca^2+^ sensors perceive [Ca^2+^]_cyt_ and form complex with kinases which further phosphorylate their downstream targets. Phosphatases dephosphorylate the same target to balance the entire mechanism. The interplay of kinases and phosphatases modulate the activity of target protein which ultimately regulates gene expression required for crop improvement (*A higher resolution / colour version of this figure is available in the electronic copy of the article*).

**Table 1:** The table represents the different components of Ca^2+^ signaling machinery and their functions in *Arabidopsis thaliana*.

**Component**	**Function**	**Refs.**
**Calcium Sensors**		
**Calmodulins**		
AtCaM1, AtCaM4	Salinity resistance by promoting accumulation of Nitric Oxide (NO)	[323]
AtCaM2	Reduces the ATP hydrolysis of DgHsp70 and its foldase activity	[324]
AtCaM3	Heat stress response	[325-327]
AtCaM4	Binds with a Sec14-like protein, PATL1 and regulates freezing tolerance	[328]
AtCaM7	Seedling development, interacts with HYPOCOTYL5 (HY5) bZIP protein to promote photomorphogenesis, associates with CNGC14 and regulates polar growth in root hairs	[30, 325, 329, 330]
**Calmodulin-Like Proteins**		
AtCML8	Defense against *Pseudomonas syringae*	[280, 331]
AtCML9	Responses to Abscisic acid (ABA), drought and salt stress, root growth in response to flagellin, herbivory stress response independent of Jasmonic acid (JA)-mediated defense responses	[250, 281, 332]
AtCML10	Regulation of Ascorbic acid synthesis	[333]
AtCML20	Negative regulation of guard cell ABA signaling under drought stress	[251]
AtCML23	Regulate NO accumulation	[334]
AtCML24	ABA-mediated inhibition of germination and seedling growth, root mechanoresponses, photoperiod-induced transition to flowering, ion homeostasis, autophagy progression through interaction with ATG4b, actin organization during pollen germination and pollen tube elongation, NO accumulation	[334-338]
AtCML25	Pollen germination and tube elongation *via* K^+^ transmembrane trafficking	[339]
AtCML36	Modulation of ACA8 activity	[340]
AtCML37	Positive regulator in Ca^2+^ signaling during herbivory through JA pathway, positive regulator of ABA under drought stress response	[284, 285]
AtCML38	Hypoxia response Ca^2+^ sensor, *A. thaliana* rapid alkalinization factor 1-mediated root growth inhibition	[341, 342]
AtCML39	Ovule and seed development, germination, light signaling during seedling establishment	[343, 344]
AtCML42	Cell branching in trichomes	[345]
AtCML43	Salicylic acid (SA)-induced root specific Ca^2+^ sensor, involved in normal growth and immune responses	[346]
**Calcineurin B-Like Proteins**		
AtCBL1	responses under salt stress, phosphate deficiency, regulation of early stress-related transcription factors of the C-Repeat-Binding Factor/dehydration-responsive element (CBF/DREB) type, CBL1-CIPK7 interactions and VDAC1-CBL1 interactions regulate cold stress responses, responses to GA and glucose during seed germination and seedling development	[308, 347-352]
AtCBL1, AtCBL9	Pollen germination and tube growth, regulation of NADPH oxidase (RBOHF) through CBL1-CIPK26 interactions	[353, 354]
AtCBL2	CBL2-CIPK6 interactions regulate sugar-homeostasis *via* sugar transporter TST2, ABA responses	[355, 356]
AtCBL2, AtCBL3	phosphorylation of CIPK9, ion homeostasis, regulates seed size and embryonic development,	[267, 357-359]
AtCBL3	CIPK9-CBL3 function under low-K^+^ stress	[360]
AtCBL4/SOS3	Salt tolerance	[361-364]
AtCBL5	Osmotic and drought stress tolerance	[365]
AtCBL7	Low nitrate response, alkali tolerance	[366, 367]
AtCBL9	Modulates ABA biosynthesis and sensitivity, transpiration and K^+^ transport in roots and guard cells, negative regulation of cold tolerance	[248, 368, 369]
AtCBL10	Salt tolerance, regulation of K^+^ homeostasis by interacting with AKT1	[265, 370-372]
**Calcium Dependent Protein Kinases**		
AtCDPK1, AtCDPK2	Induced by drought and high salt stress	[373]
AtCDPK1	Defense responses, regulates REPRESSION OF SHOOT GROWTH (RSG) transcriptional activator in GA signaling	[374, 375]
AtCDPK1/2/4/11	Phosphorylate NADPH oxidases	[300]
AtCDPK3	Enhances tolerance to Cadmium (Cd^2+^)	[376]
AtCDPK6	Phosphorylation of actin-depolymerizing factor 1 (ADF1), regulates glucose-induced stomatal closure	[377, 378]
AtCDPK4/5/6/11	Phosphorylate WRKY8/28/48 for NLR-dependent immune responses	[300]
**CBL Interacting Protein Kinases**		
AtCIPK1	Regulate root high-affinity K^+^ uptake through AtHAK5, ABA-dependent and independent stress responses, involved in the Pi deficiency signaling	[263, 379, 380]
AtCIPK3	Cold response and ABA signaling	[249, 381, 382]
AtCIPK5	Salt tolerance, wounding-induced stomatal closure through JA-activated GORK K^+^ channels	[383, 384]
AtCIPK6	Response to salt, osmotic and drought stresses, ABA, auxin transport and root growth, sugar homeostasis, negative regulation of immune response to *P. syringae*	[247, 356, 385-388]
AtCIPK7	Cold stress responses	[308]
AtCIPK8	Salt tolerance response	[372]
AtCIPK9	Low K^+^ tolerance, negative regulation of lipid accumulation, early seedling establishment, HAK5 mediated root K^+^ uptake	[256, 263, 360, 389]
AtCIPK11	Iron accumulation, negative regulator of drought stress, ABA response	[390-392]
AtCIPK12	Vacuolar Ca^2+^ signaling for polarized growth of pollen tube	[393]
AtCIPK14	Mediates WHIRLY1 (WHY1) protein deficiency in plastids, phytochrome A-mediated far-red light inhibition of seedling greening, responses to ABA and salt stress, glucose signaling	[394-397]
AtCIPK16	Salinity tolerance	[398]
AtCIPK19	Pollen tube growth and polarity	[399]
AtCIPK21	Response under osmotic, salt and cold stress	[400, 401]
AtCIPK23	Inhibits ammonium transport, regulates high-affinity K^+^ uptake in root mediated by HAK5, low K^+^ stress response by modulating AKT1, iron accumulation by modulating ferric chelate reductase activity, leaf transpiration, seed dormancy, salt and drought tolerance, primary nitrate response	[248, 262, 264, 273, 276, 299, 402, 403]
AtCIPK24	Regulation of salt tolerance	[370]
AtCIPK25	Regulation of root meristem size, modulation of K^+^ homeostasis under hypoxic conditions	[404, 405]
AtCIPK26	Involved in negative regulation of ROS with AtRbohF, modulation of RBOHC activity	[406, 407]
**Ca** ^2+^ **Transporters**		
**Cyclic Nucleotide Gated Channels**		
AtCNGC1	Growth and tolerance to Lead (Pb^2+^)	[408, 409]
AtCNGC2	Defense against pathogen, floral transition regulation, JA induction, adaptation and development of the plant under Ca^2+^ stress	[410-412]
AtCNGC3	Cation transport and seed germination	[413]
AtCNGC4	Pathogen defense, floral transition, stomatal conductance	[411, 414, 415]
AtCNGC5	Ca^2+^ influx in guard cell.	[127]
AtCNGC6	Heat shock proteins regulation and thermo-tolerance.	[127, 416]
AtCNGC7, 8	Essential for male reproductive fertility	[128]
AtCNGC9	Regulated by K^+^ starvation in roots	[417]
AtCNGC10	Negative regulator of salt tolerance *via* Na^+^ accumulation, starch accumulation and other growth responses, Ca^2+^ influx in root epidermis	[129, 418, 419]
AtCNGC11	Pathogen defense by positive regulation of SA-dependent R genes	[420]
AtCNGC12	Resistance against avirulent fungal pathogen, programmed cell death (PCD)	[420, 421]
AtCNGC14	Auxin induced Ca^2+^ signaling	[422]
AtCNGC16	Stress tolerance during pollen reproductive development.	[128]
AtCNGC17	Growth regulation along with phytosulfikine, H^+^-ATPase and BAK1	[423]
AtCNGC18	Growth of pollen tube by tip polarization phenomenon, increased Ca^2+^-conductance, stimulated by CPK32	[131, 134, 424]
AtCNGC19, 20	Salt dependent regulation.	[425]
**Glutamate Receptor-Like Channels**		
AtGLR1.1	ABA biosynthesis, control development and water loss, carbon and nitrogen metabolism	[426, 427]
AtGLR1.2	Pollen tube growth and morphogenesis by mediating Ca^2+^ influx along with *AtGLR3.7*	[109]
AtGLR1.3	Along with *AtGLR1.2,* positively regulates cold tolerance by JA signaling	[428]
AtGLR1.4	Methionine-induced membrane depolarization.	[429]
AtGLR3.1	Ca^2+^ programmed stomatal closure	[430]
AtGluR2	Unloads Ca^2+^ from the xylem vessels	[431]
AtGLR3.3	Glutathione-triggered [Ca^2+^]_cyt_ transients, innate immunity response, resistant to *Hyaloperonospora arabidopsidis*, involved in gravitropic response	[114, 432, 433]
AtGLR3.4	Seed germination under salt stress, touch and cold sensitive, regulate Ca^2+^ influx, activated by glutamate	[434, 435]
AtGLR3.5	Seed germination through GA and ROS signaling, Methionine, antagonizing the ABA inhibitory effects	[436, 437]
AtGLR3.6	Controls root development by repressing the Kip-related protein gene, *KRP4*	[111]
AtGLR3.7	Interacts with 14-3-3ω protein and salt stress response	[113]
**Two Pore Channel**		
AtTPC1	Regulation of germination, stomatal movements, K and Ca storage in the epidermal and mesophyll tissue, extracellular Ca^2+^ perception, wounding as well as JA, response in leaves to insect herbivory, response to salinity stress, may act as oxygen sensors	[137, 138, 140, 145, 148-152, 438]
**Annexins**		
AtANN1	Regulation of abiotic and biotic stress responses, Ca^2+^ signature under heat-stress, salinity stress, K^+^ and Ca^2+^ permeable conductance in root cell, post-phloem sugar transport in root tips	[153, 162, 163, 439-441]
AtANN2	Post-phloem sugar transport in root tips	[163]
AtANN4	Functions with AtANN1 in response to drought and salinity stress	[442]
AtANN5	Pollen and embryonic development	[164-166]
**Mechanosensitive-Like Channels**		
AtMSL1	Redox homeostasis maintenance	[172]
AtMSL2, AtMSL3	Regulate plastid size, shape and division growth and development, division of chloroplast	[176, 443]
AtMSL2	Regulate leaf-growth, chloroplast integrity and starch accumulation	[175]
AtMSL4,5,6,9,10	Regulate Ca^2+^ influx activated by stretching	[168]
AtMSL8	Pollen activity during hydration and germination	[178]
AtMSL10	Phosphorylation-regulated cell death	[179]
**Mid1-Complementing Activity Channels**		
AtMCA1	Ca^2+^-balance, expression of TCH3 (touch-induced CML)	[181, 182]
Hyperosmolality-gated calcium-permeable channelsAtOSCA1.1	Acts as osmosensor, DUF221 proteins (osmosensitive Ca^2+^ cation channels)	[92, 444]
**Ca**^2+^ **Exchangers**		
**Ca**^2+^**/H**^+^ **Exchangers**		
AtCAX1, AtCAX3	Ca^2+^ homeostasis, normal growth, ion homeostasis	[97, 213]
AtCAX2	Heavy metal detoxification	[445]
AtCAX3	Salt tolerance, pH sensitivity and modulate H^+^-ATPase activity, Mg^2+^-deficiency response, enhances Cd^2+^ tolerance	[214, 446, 447]
AtCAX4, AtCAX2	Root Cd sequestration	[448]
AtCAX4	Root development under metal stress	[449]
AtCAX5	Metallic stress response, Mn^2+^ transport	[450-452]
**Cation/Ca**^2+^ **Exchangers**		
AtCCX1, AtCCX4	ROS homeostasis and promote leaf senescence	[221]
AtCCX2	Osmotolerance by regulating endoplasmic reticulum and cytosolic dynamics	[222]
AtCCX3	Na^+^ and Mn^2+^ transport	[223]
AtCCX5	K^+^-uptake and regulate Na^+^ transport in yeast	[224]
**Ca** ^2+^ **-ATPase/Pumps**		
**Autoinhibited Ca**^2+^ **ATPase**		
AtACA4	Salt tolerance in yeast	[187]
AtACA7	Pollen development	[195]
AtACA8, AtACA9	Drought stress response by activating ABA signaling	[252]
AtACA10	Vegetative growth in adult plant and inflorescence formation	[196]
AtACA4, AtACA11	SA-dependent programmed cell death	[201]
AtACA12	Role in male fertility	[453]
**Endoplasmic Reticulum-Type Ca**^2+^ **ATPases**		
AtECA1	Long-distance phloem Ca^2+^ signaling in root, Mn^2+^ stress tolerance	[454, 455]
AtECA3	Regulate Manganese (Mn^2+^) nutrition	[456]

## LIST OF ABBREVIATIONS

CaM: Calmodulin
CML: CaM-like protein
CBL: Calcineurin B-like protein
CPK/CDPK: Ca^2+^-Dependent Protein Kinase
CcaMK: Ca^2+^/CaM-dependent protein kinase
CNGC: Cyclic Nucleotide-Gated Channel
GLR: Glutamate-Like Receptor
CAXs: Ca^2+^/H^+^ exchangers
TPCs: Two-Pore Channels
MSL: Mechanosensitive-Like channel
MCA: Mid1-Complementing Activity Channel
TPK: Two-Pore Potassium
GAF: cyclic GMP, adenylyl cyclase, FhlA
CNB: Cyclic Nucleotide Binding
NADK: Nicotinamide Adenine Dinucleotide Kinases
PP: Protein phosphatases
PPI: Phosphatase Interaction domain
ROS: Reactive Oxygen Species
PYR: Pyrabactin Resistance
PYL: PYR-like
SOS: Salt Overly Sensitive
ABA: Abscisic Acid
SA: Salicylic Acid
JA: Jasmonic Acid
SnRK2: Snf1-Related Protein Kinases 2
Ser: Serine
Thr: Threonine
PK: Protein Kinase
CAD: CDPK Activation Domain
CLD: CaM-Like Domain
ECA: ER-type Ca^2+^-ATPase
ACA: Autoinhibited Ca^2+^-ATPase
CAT: Catalase
SOD: Superoxide Dismutase
BiFC: Bimolecular Fluorescence Complementation
FRET: Fluorescence Resonance Energy Transfer
